# Zonated Copper‐Driven Breast Cancer Progression Countered by a Copper‐Depleting Nanoagent for Immune and Metabolic Reprogramming

**DOI:** 10.1002/advs.202412434

**Published:** 2025-04-24

**Authors:** Lin Chen, Saibo Ma, Hao Wu, Lingna Zheng, Yunpeng Yi, Guangnian Liu, Baoyi Li, Jiayi Sun, Yang Du, Bing Wang, Yike Liu, Cheng Zhang, Jing Chang, Yuheng Pang, Wenjing Wang, Meng Wang, Motao Zhu

**Affiliations:** ^1^ CAS Key Laboratory for Biomedical Effects of Nanomaterials & Nanosafety CAS Center for Excellence in Nanoscience National Center for Nanoscience and Technology Beijing 100190 China; ^2^ School of Nanoscience and Engineering University of Chinese Academy of Science Beijing 100049 China; ^3^ College of Marine Life Science Ocean University of China Qingdao 266003 China; ^4^ Key Laboratory of Nuclear Analytical Techniques and Key Laboratory for Biomedical Effects of Nanomaterials and Nanosafety Institute of High Energy Physics Chinese Academy of Sciences Beijing 100049 China; ^5^ Shandong Provincial Animal and Poultry Green Health Products Creation Engineering Laboratory Institute of Poultry Science Shandong Academy of Agricultural Science Jinan 250100 China; ^6^ Department of Hepatobiliary and Pancreatic Surgery Peking University First Hospital Beijing 100035 China; ^7^ State Key Laboratory of Holistic Integrative Management of Gastrointestinal Cancers Beijing Key Laboratory of Carcinogenesis and Translational Research Department of Gastrointestinal Oncology Peking University Cancer Hospital & Institute Beijing 100142 China; ^8^ Beijing YouAn Hospital Capital Medical University Beijing Institute of Hepatology Beijing 100069 China

**Keywords:** *Akkermansia muciniphila*, breast cancer, copper, metabolic reprogramming, outer membrane vesicles, spatially resolved multiomics, tetrathiomolybdate

## Abstract

While studies of various carcinomas have reported aberrant metal metabolism, much remains unknown regarding their spatial accumulation and regulatory impacts in tumors. Here, elevated copper levels are detected in breast cancer tumors from patients and animal models, specifically exhibiting a zonate spatial pattern. Spatially resolved multiomics analyses reveal that copper zonation drives a tumor metabolic preference for oxidative phosphorylation (OXPHOS) over glycolysis and promotes tumor metastatic and immune‐desert phenotypes. Then, a copper‐depleting nanoagent is developed based on copper chelator tetrathiomolybdate (TM)‐loaded hybridized bacterial outer membrane vesicles (hOMVs) from both *Akkermansia muciniphila* bacteria and CD326‐targeting peptide‐engineered *Escherichia coli* (TM@^CD326^hOMV). Systemic administration of TM@^CD326^hOMV reduces the labile copper level in tumors and inhibits both tumor growth and metastatic phenotypes, specifically through metabolic reprograming of OXPHOS toward glycolysis and restoration of antitumor immunity responses involving natural killer cells, CD4^+^ T cells, and cytotoxic CD8^+^ T cells in tumors. Assessing survival in murine breast cancer models, a combination of TM@^CD326^hOMV and a checkpoint blockade agent outperforms monotherapies. Notably, a copper‐rich diet undermines the therapeutic efficacy of TM@^CD326^hOMV. Beyond demonstrating an effective nanoagent for treating breast cancer, this study deepens the understanding of how the pattern of copper accumulation in tumors affects pathophysiology and immunity.

## Graphical abstract

Spatially resolved multiomics reveals that high‐copper zones are closely associated with tumor metastasis, immune‐desert phenotypes, and oxidative‐phosphorylation (OXPHOS)‐dependent proliferation. A targeted bacterial nanovesicle delivering copper‐depleting agents effectively reduces tumor copper levels, inhibits OXPHOS‐dependent tumor growth, and enhances antitumor immunity. This strategy provides a promising approach to cancer immunotherapy and enhances the effectiveness of immune checkpoint inhibitors.

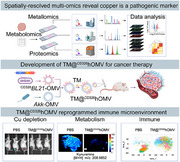

## 1 Introduction

Metals are essential for myriad biological processes, including participation in enzymatic reactions, redox regulation, and signal transduction.^[^ ^1^ ^]^ In the context of cancer, distinct metals (and combinations) have been shown to profoundly influence cancer initiation, progression, drug resistance, and immunity.^[^ ^2^ ^]^ Altered metal levels, for example, elevated iron and copper, are frequently observed in patient tumors^[^ ^3^ ^]^ and are associated with elevated metastasis risk and poor prognosis.^[^ ^4^ ^]^ Despite these associations suggesting that metal accumulation may influence tumor evolution and affect therapeutic outcomes, the distribution of metals within the heterogeneous tumor microenvironment, as well as their interaction with key metabolic and immunological pathways, remains largely unexplored.

Recent discoveries have highlighted the role of copper in regulating pathways such as mitochondrial respiration, antioxidant defense, and inflammation.^[^ ^5^ ^]^ Dysregulated copper metabolism can drive copper‐dependent cell growth and proliferation (so‐called “cuproplasia”) and regulatory cell death (“cuprotosis”).^[^ ^6^ ^]^ Copper serves as a cofactor for cytochrome C oxidase (also referred to as complex IV of the mitochondrial electron transport chain), which functions in cellular respiration and energy production.^[^ ^7^ ^]^ In certain cancers that are less sensitive to glycolysis inhibition therapy, such as triple‐negative breast cancer (TNBC), mitochondrial copper depletion has been shown to significantly inhibit copper‐dependent mitochondrial oxidative phosphorylation (OXPHOS) and tumor growth.^[^ ^8^ ^]^ Copper also exerts a regulatory role in angiogenesis and cancer immunity by modulating the expression of programmed cell death protein 1 (PD‐1) and macrophage polarization.^[^ ^5a^ ^]^ As a double‐edged sword to cancer, a more sophisticated understanding of how copper is harnessed in tumor metabolism, metastasis, and immune surveillance is imperative. Gaining such knowledge would expedite the development of tailored therapies using existing copper‐regulatory pharmacological agents, such as copper chelators and copper ionophores,^[^ ^8^, ^9^ ^]^ to treat cancer and in combination with other antitumor modalities.

Leveraging the spatially resolved multiomics technologies, we first profiled the tumor metallomic landscape of breast tumor tissues from patients and various mouse models using laser ablation with inductively coupled plasma mass spectrometry (LA‐ICP‐MS) and discovered copper accumulation in breast tumors with a zonate spatial distribution pattern. By integrating spatially resolved spatial metabolomics and proteomics, we found that the high‐copper zones of tumors favor OXPHOS over glycolysis, and display metastasis and immunosuppressive characteristics. Taking cues from these findings, we developed a copper‐depleting nanoagent using hybrid bacterial outer membrane vesicles (^CD326^hOMVs) derived from both CD326‐targeting peptide‐engineered *Escherichia coli* and next‐generation probiotic *Akkermansia muciniphila* (*Akk*).^[^ ^10^ ^]^ Outer membrane vesicles (OMVs), particularly those derived from *Akk*, are naturally enriched with pathogen‐associated molecular patterns (PAMPs), possess inherent immunomodulatory functions.^[^ ^10^, ^11^ ^]^ Therefore, OMVs serve as versatile platforms for small molecule drug delivery and bioactive vesicles for tumor immune microenvironment reprograming. After generating copper chelator tetrathiomolybdate (TM)‐loaded ^CD326^hOMVs (TM@^CD326^hOMV), we treated 4T1 breast tumor models with this nanoagent and found that the targeted elimination of labile copper significantly inhibited tumor growth. Specifically, treatment with TM@^CD326^hOMV resulted in a metabolic shift from OXPHOS to glycolysis within the tumors, a reduction in metastatic phenotypes (e.g., lysyl oxidases (LOX), collagen I, vimentin), and an increase in natural killer (NK) cells, CD4^+^ T cells, and cytotoxic CD8^+^ T cells. These features of TM@^CD326^hOMV contributed to an improved response to anti‐PD‐1 antibody therapy in both 4T1 and epithelial‐to‐mesenchymal transition (EMT)‐6 breast cancer mouse models. Notably, a high‐copper diet significantly negated the antitumor effects of both monotherapy and combination therapy, highlighting that medical supervision of copper intake is pertinent when targeting copper regulation for breast cancer therapy.

## 2 Results

### 2.1 Copper Is Accumulated and Spatially Segregated in Breast Tumor Tissues from Patients and Multiple Animal Models

**Figure 1:**
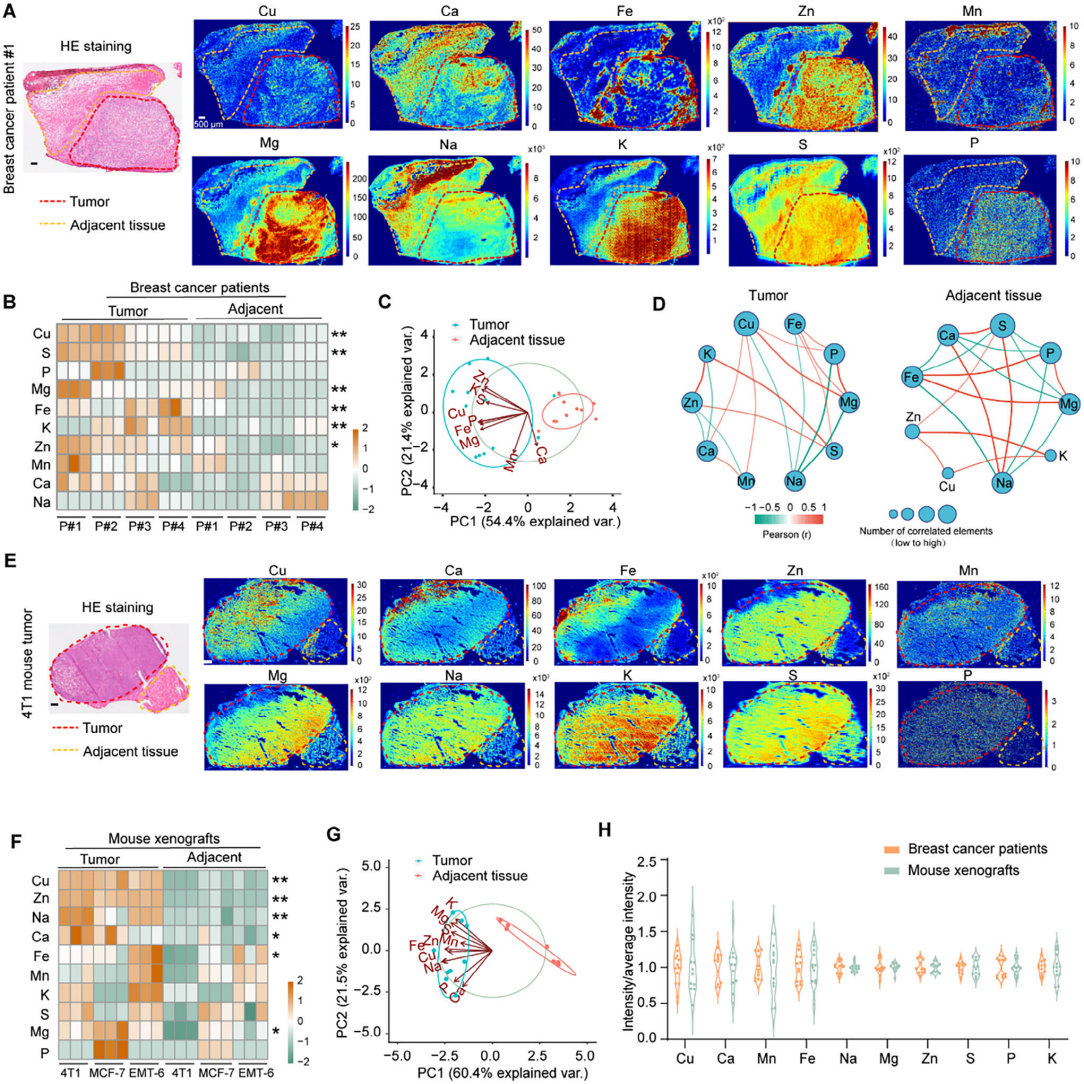
Copper is accumulated and spatially segregated in breast tumor tissues. A) Representative LA‐ICP‐MS images of the metallome of the tumor and adjacent tissue in sections of a resected human breast cancer tumor. The hydrogel‐embedded sections were analyzed for copper (^63^Cu), calcium (^44^Ca), iron (^56^Fe), zinc (^64^Zn), manganese (^55^Mn), magnesium (^24^Mg), sodium (^23^Na), potassium (^39^K), sulfur (^32^S), and phosphorus (^31^P). Scale bar: 500 µm. B) Heatmap depicting the metallome of breast cancer and adjacent tissues from breast cancer patients (3 ROIs were analyzed for each patient, *n* = 4). C) Principal component analysis (PCA) of the metal concentration of the tumors and adjacent tissues of breast cancer patients. D) Network of Pearson correlation coefficients between metals. The correlation coefficient and directionality of the correlation are pictured through line thickness and color. Node size indicates the number of correlated elements. Only Pearson coefficients greater than 0.5 are presented. E) Representative LA‐ICP‐MS images of the metallome of tumor and adjacent tissue in 4T1 breast cancer mouse xenografts. F) Heatmap depicting the metallome of breast cancer and adjacent tissues from 4T1, MCF‐7, and EMT‐6 breast cancer mouse models (3 representative samples for each tumor type). G) PCA of metals calculated in panel (F) in three mouse models. H) Heterogeneity of metals assessed by intensity across 12 regions, dividing by the average intensity of each sample (*n* = 3). *p* values were obtained using unpaired two‐tailed *t*‐tests in (B) and (F). *p < 0.05; **p < 0.01; **p < 0.001.

Given the reported but poorly understood dysregulation of various metals in tumors, we sought to gain a finer‐grain understanding of tumor metal distributions. We adopted a high‐resolution analytical method to profile resected tumors and adjacent samples from 4 breast cancer patients (**Table** **1**), particularly using LA‐ICP‐MS to resolve samples at a 10 µm scale. We found that the levels/concentrations of several transition trace metals, known to exist primarily in the form of protein complexes^[^ ^12^ ^]^ [e.g., Cu, zinc (Zn), and iron (Fe)], were consistently significantly higher in tumors than in adjacent tissues, based on the calculation of the signal intensity of randomly selected 3 regions of interests (ROIs, 1 mm^2^ of each) in each sample (**Figures** **1**A,B and S1 (Supporting Information)), regardless of the clinical subtype (Table 1). We also detected significantly higher levels of the alkali metal potassium (K) and the alkaline earth metal magnesium (Mg) in tumors than in adjacent tissues (Figures 1A,B and S1 (Supporting Information)).

**Table 1:** Clinical data for breast cancer patients. ER, estrogen R∖receptor; HER2, human epidermal growth factor receptor 2; TNBC, triple‐negative breast cancer.

	Gender	Age	Race	TNM stage	Histology subtype	Clinical subtype
Patient 1	Female	88	Asia	III	Invasion ductal carcinoma	ER^+^/HER2^+^
Patient 2	Female	68	Asia	II	Invasion ductal carcinoma	HER2^+^
Patient 3	Female	55	Asia	II	Invasion ductal carcinoma	ER^+^
Patient 4	Female	58	Asia	II	Invasion ductal carcinoma	TNBC

Consistent with the previously recognized aberrant metal metabolism in tumor,^[^ ^13^ ^]^ tumor tissues and the adjacent tissues display distinct metallomes as revealed by principal component analysis (PCA) (Figure 1C). Pearson's correlation analysis revealed that copper in tumor tissues positively correlates (Pearson's *r* > 0.5) with Mg, calcium (Ca), manganese (Mn), and phosphorus (P), yet negatively correlates (Pearson's *r* < −0.5) with sodium (Na) (Figure 1D). By contrast, Zn and K positively correlate with copper in adjacent tissues, and no correlations within the defined cutoffs were detected for the other metals [Na, Mg, Ca, Fe, P, and sulfur (S)]. These findings suggest that elevated levels of transition metals, particularly copper, interact dynamically with other metals to support metabolic activities in tumors.

Having detected consistently evident trends for tumor‐specific metal accumulation in resected human patient samples, we next examined whether such tumor‐specific accumulation is evident in a diverse set of mouse breast cancer models, including 4T1 murine TNBC tumors, EMT‐6 murine TNBC tumors, and MCF‐7 human TNBC tumors (Figure 1E,F and Figure S2 (Supporting Information)). We resected the murine tumors and adjacent tissues and conducted LA‐ICP‐MS. As in the human patient samples, we observed obvious differences in metal accumulation in tumors versus adjacent tissue for the three examined tumor types, including tumor accumulation for Cu, Zn, Ca, Fe, Na, and Mg (Figure 1E–G).

By further zooming into the spatial distribution of metals, the dispersion index (variance to mean ratio) calculated using 12 selected ROIs (4 × 1 mm^2^ of each sample) revealed a highly spatially segregated accumulation pattern for metals including Cu, Fe, and Ca in breast tumor tissues from both patient samples and 4T1 murine models (Figure 1H); such patterns were not detected for Na, Mg, Zn, K, S, or P. Notably, peripheral blood samples of the 4T1 tumor group mice had significantly higher plasma copper concentrations than nonmodel control mice, suggesting an increased demand for copper (Figure S3A,B, Supporting Information). Ultimately, this increased copper demand and the highly spatially segregated accumulation pattern for copper (in both human and murine model tumors) motivated a follow‐up analysis of the potential biological contributions of copper in tumor subregions.

### 2.2 High‐Copper Zones of Tumors Exhibit Efficient OXPHOS Metabolism and Both Metastatic and Immune‐Desert Phenotypes

**Figure 2:**
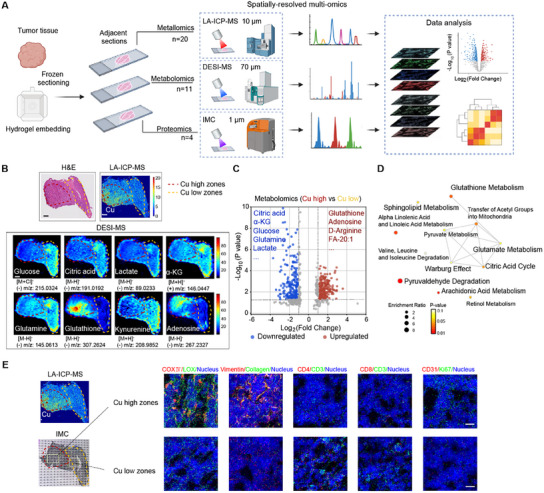
High‐copper zones of tumors exhibit efficient OXPHOS metabolism and both metastatic and immune‐desert phenotypes. A) Schematic representation of a spatial multiomics analysis examining the spatial distribution of metals, metabolites, and proteins within the 4T1 tumor. B) Representative LA‐ICP‐MS, DESI‐MS, and histological (H&E) staining images showing the spatial distribution of copper and the indicated metabolites in a 4T1 tumor (the color scales of intensity in MS images are for relative values). Scale bar: 500 µm. C) Volcano plots illustrating the differential abundance of metabolites in high‐copper versus low copper zones within the tumor (*n* = 8). D) Network visualization depicting the significantly enriched metabolic pathways in the high‐copper zones relative to the low copper zones. E) Imaging mass cytometry (IMC) images used for assessing the distribution of CD3, CD4, CD8, COX_IV, LOX, vimentin, and Ki67 in high‐ and low‐copper zones in the same sample shown in panel (B). Representative images are shown from four independent samples. Scale bar: 20 µm.

By integrating LA‐ICP‐MS imaging for the tumor metallome, desorption electrospray ionization mass spectrometry imaging (DESI‐MSI) for spatial metabolomics (with 70 µm resolution), and imaging mass cytometry (IMC) for spatial proteomics (with 1 µm resolution), we analyzed potential relationships between copper and various metabolites and proteins in serial sections of 4T1 tumors (**Figure** **2**A). Strikingly, the particular zonate distribution of copper aligned well with the observed distribution of multiple metabolites, including organic acids and sugars of carbohydrate metabolism and precursors of amino acid synthesis (Figure 2B).

Upon dividing tumors into Cu‐high and Cu‐low zones (based on the median value of copper intensity), an orthogonal partial least squares discrimination analysis (OPLS‐DA) model indicated distinct metabolite distribution patterns between the two regions (Figure S3C, Supporting Information). Specifically, the high‐Cu zones were deficient in glucose, glutamine, citric acid, and lactate, but enriched for glutathione, adenosine, arginine, and fatty acids (FA)‐20:1 (Figure 2B,C), as compared to the low‐Cu zones. The low level of lactate (known as the end product of glycolysis^[^ ^14^ ^]^) indicated glycolytic suppression in the high‐Cu zones. A metabolite set enrichment analysis (MSEA) comparing the high‐ and low‐Cu zones indicated enrichment for pathways including pyruvaldehyde degradation, the citric acid cycle, the Warburg effect, glutamate metabolism, and glutathione metabolism in high‐Cu zones (Figure 2D), suggesting that copper distribution drives OXPHOS‐favored metabolism in tumors in a zonate pattern.

Given the previously reported effects of the OXPHOS–glycolysis axis, glutamine, fatty acids, and adenosine in immunometabolic reprogramming,^[^ ^15^ ^]^ we next assessed tumor immune phenotypes in Cu‐high and Cu‐low zones. By labeling selected proteins with metal‐reporter‐conjugated antibodies and analyzing selected ROIs (1 mm × 1 mm) in high‐Cu and low‐Cu zones and collecting IMC data, we detected obvious enrichment of cuproenzymes (e.g., complex IV) in high‐Cu zones. Additionally, tumor immune markers for infiltrating T cells (e.g., CD3, CD4, and CD8) were detected at obviously lower levels in the high‐Cu zones than in low‐Cu zones (Figure 2E), suggesting an immune‐desert phenotype (characterized by a paucity of T cells in either the parenchyma or the stroma of the tumor^[^ ^16^ ^]^) in the high‐Cu zones. Intriguingly, this analysis also revealed enrichment in high‐Cu zones for multiple known tumor metastatic markers, including LOX (copper‐dependent enzymes as well as a tumor migration marker), vimentin, and collagen I (Figure 2E). Extracellular matrix (ECM) plays a critical role in cancer progression. We analyze the correlation of metal and ECM components, as illustrated in the heatmap (Figure S4A, Supporting Information). Notably, LOX1, a key enzyme in ECM remodeling,^[^ ^17^ ^]^ exhibited a strong positive correlation with copper and manganese, suggesting that copper may promote ECM remodeling through LOX1‐mediated collagen cross‐linking and ECM stiffness. Vimentin, a marker of EMT,^[^ ^18^ ^]^ was significantly associated with manganese, indicating a potential link between manganese levels and EMT‐driven ECM reorganization. Additionally, collagen I, a major structural component of ECM,^[^ ^19^ ^]^ exhibits a significant correlation with zinc, implying that zinc may contribute to ECM structural organization. Collectively, the copper‐zonation‐related tumor hallmarks suggest that high‐Cu zones are particularly pathogenic (relative to the low‐Cu zones) in terms of malignancy, cancer proliferation, and immune‐desert phenotype.

### 2.3 TM@^CD326^hOMV Targets Tumors and Durably Depletes Labile Copper

**Figure 3:**
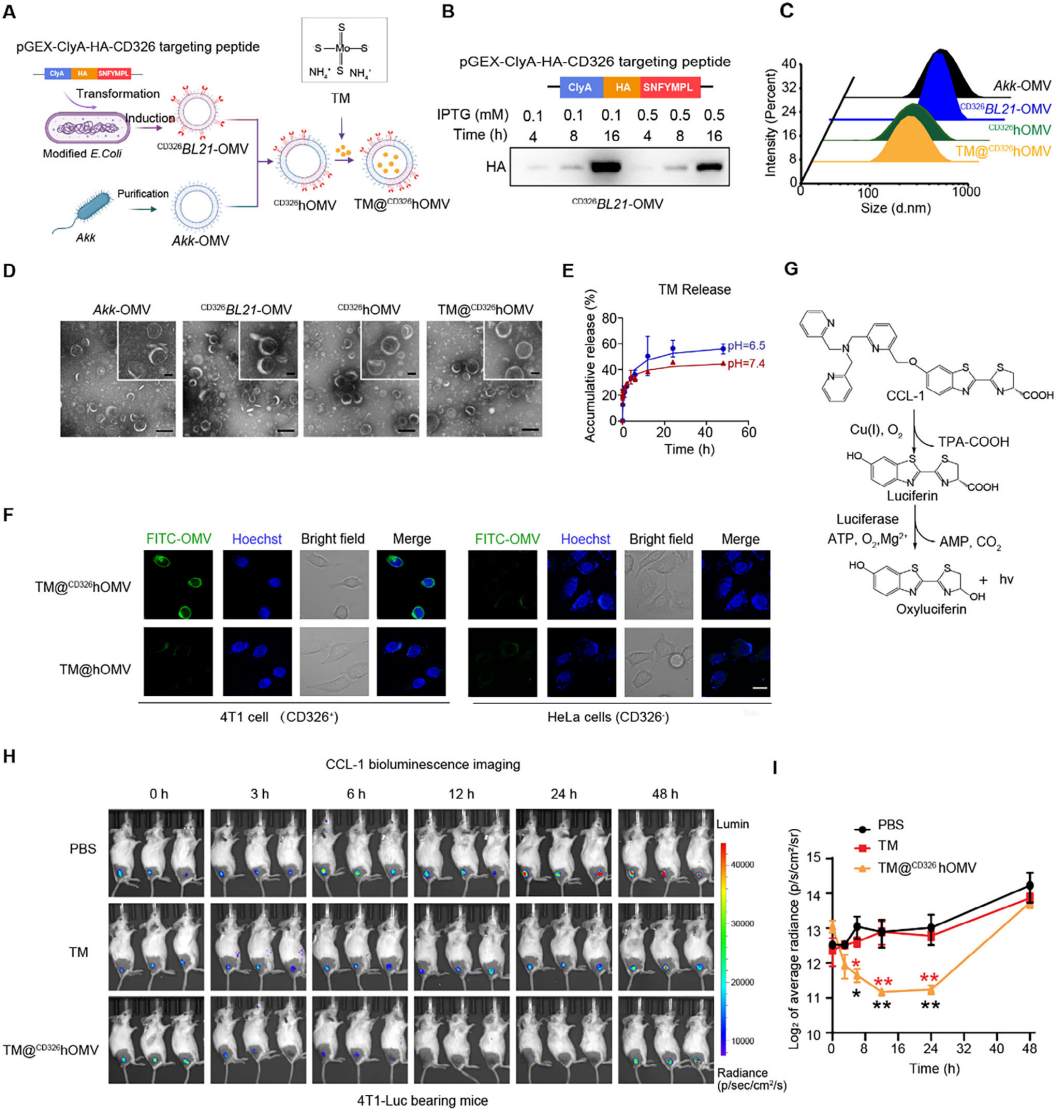
Development and characterization of TM@^CD326^hOMV for targeted depletion of labile copper. A) Schematic illustration of the fabrication process of TM@^CD326^hOMV, involving the isolation of ^CD326^ *BL21*‐OMV from genetically modified *BL21* bacteria expressing a ClyA–HA–CD326‐targeting peptide, followed by hybridization with *Akk*‐OMV via ultrasonication and extrusion, and TM loading via electroporation. B) Immunoblotting for the presence of the CD326‐targeting peptide on ^CD326^ *BL21*‐OMV using an anti‐HA antibody. C) Size distribution of indicated vesicle types measured by dynamic light scattering. D) Representative transmission electron microscopy (TEM) images of *Akk*‐OMV, ^CD326^ *BL21*‐OMV, ^CD326^hOMV, and TM@^CD326^hOMV. Scale bar: 200 nm. E) In vitro release kinetics of TM from TM@^CD326^hOMV at pH 7.4 or 6.5 (PBS containing 0.1% Tween‐20). F) Confocal microscopy images of 4T1 (CD326^+^) and HeLa (CD326^−^) cells after incubation with FITC‐labeled TM@^CD326^hOMV or TM@hOMV at 4 °C for 1 h. Cell nuclei (blue) were stained with Hoechst 33342. Scale bar: 20 µm. G) Working principle for use of a copper‐caged luciferin‐1 (CCL‐1) probe to image labile copper. CCL‐1 is a TPA‐ligand‐caged probe that releases d‐luciferin upon oxidative cleavage with Cu^+^ for a subsequent bioluminescence reaction with firefly luciferase. H) Bioluminescence images of 4T1‐Luc tumor‐bearing mice post‐i.v.‐injection of PBS, TM, or TM@^CD326^hOMV. Mice were intraperitoneally injected with CCL‐1 (6 mg kg^−1^) prior to IVIS spectrum imaging at each time point. I) Quantification of bioluminescence intensities of luciferase in different groups at the indicated times. Data are presented as the mean ± SD (*n* = 3). *p* values were obtained using unpaired two‐tailed *t*‐tests. *p < 0.05; **p < 0.01.

We envisioned a nanomedicine intervention targeting tumor‐resident labile copper with the aim of immunometabolic reprogramming as an antitumor measure. We designed a tumor‐targeted hybridized bacterial OMV from both *Akk* and CD326‐targeting peptide‐engineered *E. coli* bacteria for the delivery of a copper chelator (**Figure** **3**A).

CD326, also known as epithelial cell adhesion/activating molecule (EpCAM),^[^ ^20^ ^]^ is expressed at high levels in human breast cancers^[^ ^21^ ^]^ as well as 4T1, EMT‐6, and MCF‐7 cells (Figure S5A, Supporting Information). We generated an *E. coli BL21* bacterium displaying a CD326‐targeting peptide (SNFYMPL) (^CD326^ *BL21*) by transforming an isopropyl‐β‐d‐thiogalactoside (IPTG)‐inducible plasmid encoding a fusion protein comprising cytolysin A (ClyA) protein and HA‐tagged SNFYMPL for expression on the bacterial outer membrane surface. Testing identified the optimal expression conditions as 16 °C for 16 h using 0.01 mm IPTG induction (Figure 3B). Bacterial OMVs are enriched of an abundance of PAMPs and exhibit immunomodulation ability. In particular, *Akk*‐derived OMV was selected owing to its known capacity to activate CD8^+^ T cell responses and to recruit tumor‐killing M1 macrophages.^[^ ^22^ ^]^ OMVs from wild‐type *Akk* and ^CD326^ *BL21* were hybridized into ^CD326^hOMV by mixing ^CD326^ *BL21*‐OMV and *Akk*‐OMV at a mass ratio of 1:2, followed by emulsification and serial extrusion. The fusion of the two individual OMVs was measured by a fluorescence resonance energy transfer assay. The successful fusion was evident by the superposition effect of the fluorescent emission, where the physical mixture of the DiL‐labeled ^CD326^ *BL21*‐OMV and DiO‐labeled *Akk*‐OMV served as a control (Figure S4B,C, Supporting Information). To profile the proteins comprising the ^CD326^hOMV, we resolved the proteins by sodium dodecyl sulfate–polyacrylamide gel electrophoresis and stained the resulting gels with Coomassie brilliant blue. The protein profile of ^CD326^hOMV resembled a combination of proteins from the two individual OMVs (*Akk‐*OMV and ^CD326^ *BL21*‐OMV) (Figure S4D, Supporting Information). The prepared ^CD326^hOMV was stable at 4 and at 37 °C for one week, as assessed using dynamic light scattering (DLS) (Figure S4E, Supporting Information). *Akk*‐OMV, ^CD326^ *BL21*‐OMV, ^CD326^hOMV, and TM@^CD326^hOMV all exhibit typical cup‐shaped, bilayered membranous morphologies with mean diameters of 110–190 nm based on transmission electron microscopy (TEM) observation and DLS analysis (Figure 3C,D). The surface membranes of all OMVs are negatively charged (Figure S4F, Supporting Information).

To demonstrate the necessity of incorporating *Akk*‐OMV, we first evaluated the ability of OMVs to activate and mature BMDCs. BMDCs were treated with ^CD326^ *BL21*‐OMV or ^CD326^hOMV, respectively. We found that ^CD326^hOMV induced significantly higher levels of proinflammatory cytokine productions (TNF‐α, IL‐1β, and IL‐6) compared to ^CD326^ *BL21*‐OMV after 12 h of incubation. Additionally, ^CD326^hOMV stimulation led to a greater upregulation of maturation markers (CD80: 59.6% vs 51.2%; CD86: 59.6% vs 50.8%) and antigen‐presenting molecules (MHC‐I: 49.1% vs 44.6%; MHC‐II: 62.7% vs 50.5%) compared to ^CD326^ *BL21*‐OMV (Figure S5A–D, Supporting Information). To evaluate the potential of OMVs in adjuvating antigen‐specific CD8^+^ T cell activation, we isolated OVA^257–264^‐specific CD8^+^ T cells from OT‐1 mouse spleens and cocultured them with BMDCs in the presence of OVA^257–264^ peptide, along with either ^CD326^ *BL21*‐OMV or ^CD326^hOMV (Figure S5E, Supporting Information). ^CD326^hOMV stimulation induced significantly higher IFN‐γ production (680 pg mL^−1^) compared to ^CD326^ *BL21*‐OMV (360 pg mL^−1^) (Figure S5F, Supporting Information). These results collectively demonstrate that incorporating *Akk*‐OMV significantly enhances both DC maturation and subsequent CD8^+^ T cell activation compared to ^CD326^ *BL21*‐OMV, underscoring the necessity of using *Akk*‐OMV.

TM, a FDA‐approved copper selective chelator for mitigating conditions associated with copper overload such as Wilson's disease and certain types of cancer,^[^ ^23^ ^]^ was subsequently loaded into ^CD326^hOMV via electroporation. The loading efficiency of TM was ≈29%, calculated based on specific absorbance at 468 nm (Figure S6A, Supporting Information). At physiological pH 7.4, the cumulative release of TM from the ^CD326^hOMV was about 45% within 48 h; at a pH of 6.5, the cumulative release of TM reached ≈56% within 48 h (Figure 3E).

To assess the specific interaction of TM@^CD326^hOMV with tumor cells in vitro, 4T1 cells (CD326^+^) and HeLa cells (CD326^−^) were incubated with fluorescein isothiocyanate (FITC)‐labeled TM@^CD326^hOMV or TM@hOMV at 4 °C. TM@^CD326^hOMV adhered to the 4T1 cell membrane more efficiently than HeLa cells based on confocal microscope observation of the FITC signals surrounding the 4T1 cells (CD326^+^). TM@^CD326^hOMV adhered to the cell membrane more efficiently than TM@hOMV (Figure 3F), suggesting a specific interaction between TM@^CD326^hOMV and the CD326^+^ cell membrane. For the cellular uptake analysis, 4T1 cells were treated with FITC‐labeled OMVs at 37 °C for 1 and 3 h. Flow cytometry analysis revealed that FITC‐labeled TM@^CD326^hOMV exhibited a 5.5‐fold increase in cellular uptake by 4T1 cells compared to TM@hOMV after 3 h of incubation (Figure S6B, Supporting Information). We also measured the in vivo biodistribution of TM@^CD326^hOMV after intravenous injection of Cy5.5‐labeled TM@^CD326^hOMV in 4T1 tumor‐bearing mice. The Cy5.5 signal was detectable at the tumor site starting from 1 h postinjection and reached its peak concentration at 24 h (Figure S7A, Supporting Information). The signals persisted beyond the 48 h observation time point, indicating the efficient trafficking and accumulation of TM@^CD326^hOMV in tumor (Figure S7A, Supporting Information). At 24 h postinjection, tumors and major organs were imaged ex vivo; strong fluorescence signals were observed for the tumor tissue as well as the liver, spleen, and kidney (Figure S7B, Supporting Information).

Copper exists in Cu^+^ and Cu^2+^ states, with Cu^+^ being predominant in the reducing environment of the cytoplasm and considered the “labile” form for numerous physiological processes.^[^ ^9^ ^]^ To evaluate the elimination of tumor resident labile copper by TM@^CD326^hOMV, we first synthesized a bioluminescent probe known as copper‐caged luciferin‐1 (CCL‐1).^[^ ^24^ ^]^ Upon oxidative cleavage with Cu^+^, CCL‐1 releases d‐luciferin, which is exploited in a subsequence reporter reaction with firefly luciferase (Figure 3G). The intensity of the firefly luciferase is positively related to the copper concentration (Figure S8A,B, Supporting Information). To test the cellular uptake and copper depletion, we coincubated 4T1‐Luc cells with TM@^CD326^hOMV and different concentrations of copper. The addition of TM@^CD326^hOMV decreased the labile copper concentration in a sustained manner as revealed by a continuously decreasing bioluminescent signal from CCL‐1 till 24 h postincubation, indicating a sustained release of TM from TM@^CD326^hOMV (Figure S8C,D, Supporting Information). By contrast, the bioluminescent signal of the TM group decreased sharply at 3 h postincubation and gradually increased to a significantly higher level than that of the TM@^CD326^hOMV at 24 h (Figure S8C,D, Supporting Information). To study the copper‐depleting dynamics in vivo, we monitored CCL‐1 bioluminescence in 4T1‐Luc tumor‐bearing mice after a single intravenous injection of TM@^CD326^hOMV. The labile copper level depicted by CCL‐1‐enabled bioluminescence consistently showed a sustained decrease in tumors for 24 h (Figure 3H,I). By contrast, the administration of solitary TM did not lead to any alteration in labile copper levels in the tumor at any examined time points (Figure 3H,I). These findings collectively demonstrate the efficient trafficking of TM@^CD326^hOMV to CD326‐expressing tumors to deplete labile copper in tumors.

### 2.4 TM@^CD326^hOMV Induces Potent Antitumor Effects in a Copper‐Dependent Manner

**Figure 4:**
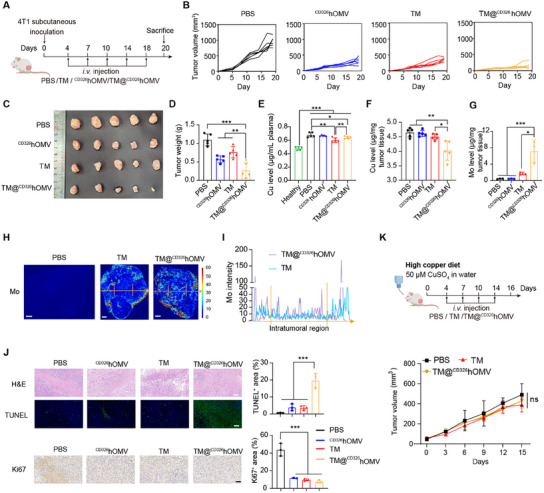
TM@^CD326^hOMV induces a strong antitumor effect in a copper‐dependent manner. A) Experimental design schematic. Female BALB/c mice were subcutaneously inoculated with 4T1 cells (2 × 10^6^ per mouse), followed by tail vein injections of PBS, TM (25 µg per mouse), ^CD326^hOMV (60 µg per mouse), or TM@^CD326^hOMV (60 µg protein containing 25 µg TM per mouse) on days 4, 7, 10, 14, and 18 (a total of 5 times). B) Tumor growth curves for each individual mouse in the indicated treatment groups. C,D) Tumors (C) and average tumor weights (D) at the endpoint (day 20) of the experiment. Data are represented as the mean ± SD (*n* = 5) from three independent experiments. E,F) ICP‐MS quantification of the copper content in plasma (E) and tumor tissue (F). Data are represented as the mean ± SD (*n* = 5). G) Quantification of molybdenum (^98^Mo) content in tumor tissue via ICP‐MS analysis. Data are represented as the mean ± SD (*n* = 5). H,I) Representative LA‐ICP‐MS images of ^98^Mo in tumors from 3 independent mice of each group (H). Scale bar, 500 µm. ^98^Mo intensity in tumors treated with TM or TM@^CD326^hOMV were analyzed across the intratumoral region (yellow arrows) (I) (*n* = 3). J) H&E images, TUNEL staining, and Ki67 immunohistochemistry in tumor tissues from the indicated groups. Quantification of TUNEL and Ki67 positive areas is shown in the right panel. Scale bar, 100 µm. Data are represented as the mean ± SD (*n* = 3). K) Experimental design schematic (upper panel) and antitumor effect of TM@^CD326^hOMV and TM in mice receiving high‐copper water‐feeding (containing 50 µm CuSO_4_). Data are represented as the mean ± SD (*n* = 5). *p* values were determined by one‐way ANOVA with Tukey's multiple comparisons test in (D–G) and (J). NS, not significant. *p < 0.05; **p < 0.01; ***p < 0.001.

We next investigated the in vivo therapeutic efficacy of TM@^CD326^hOMV in a 4T1 subcutaneous tumor mouse model (**Figure** **4**A). Receiving a total of 5 doses of i.v. injection (interval of 3–4 days), tumors in the TM@^CD326^hOMV group exhibited significant inhibition compared to the TM and ^CD326^hOMV groups (Figure 4B–D). We proceed to determine the plasma levels and distribution of copper and TM (represented by molybdenum, Mo) levels in the tumor tissues using ICP‐MS and LA‐ICP‐MS. Plasma copper levels in TM@^CD326^hOMV‐treated mice were reduced to a weaker extent than in the free‐TM‐treated mice (Figure 4E). Notably, copper levels in tumors were significantly reduced after TM@^CD326^hOMV treatment compared to the vehicle control group and compared to mice given free TM (Figure 4F). ICP‐MS analyses revealed a 3.2‐fold increase in TM (Mo) levels in resected tumor tissues from the TM@^CD326^hOMV group compared to the free TM group (Figure 4G), suggesting that ^CD326^hOMV delivery enabled significant accumulation of TM in tumors. We further examined the distribution of molybdenum (^98^Mo) in tumors using LA‐ICP‐MS and found noticeable higher TM levels in deep tumors compared to free TM, as indicated by the Mo signal along the yellow arrows across the intratumoral region (Figure 4H,I). Elimination of intratumoral copper significantly inhibited tumor proliferation (as indicated by Ki67^+^ cells) and increased tumor apoptosis (as indicated by a significant TUNEL‐positive signal) in the TM@^CD326^hOMV group compared to the phosphate‐buffered saline (PBS) or free TM group (Figure 4J). Notably, by introducing a high‐copper diet (50 µm CuSO_4_ in drinking water) during the therapy, the antitumor effect of TM@^CD326^hOMV was completely undermined (Figure 4K).

No differences in mouse body weight were detected among groups throughout the experiment, nor were there changes in serum biochemical biomarkers (e.g., alanine transaminase, aspartate transaminase, or blood urea nitrogen) or in histopathological features of major organs (Figure S9A–E, Supporting Information), suggesting no obvious systemic toxicity of TM@^CD326^hOMV treatment.

### 2.5 TM@^CD326^hOMV Reprograms the Tumor Metabolism and Restores Antitumor Immunity in Breast Tumors

**Figure 5:**
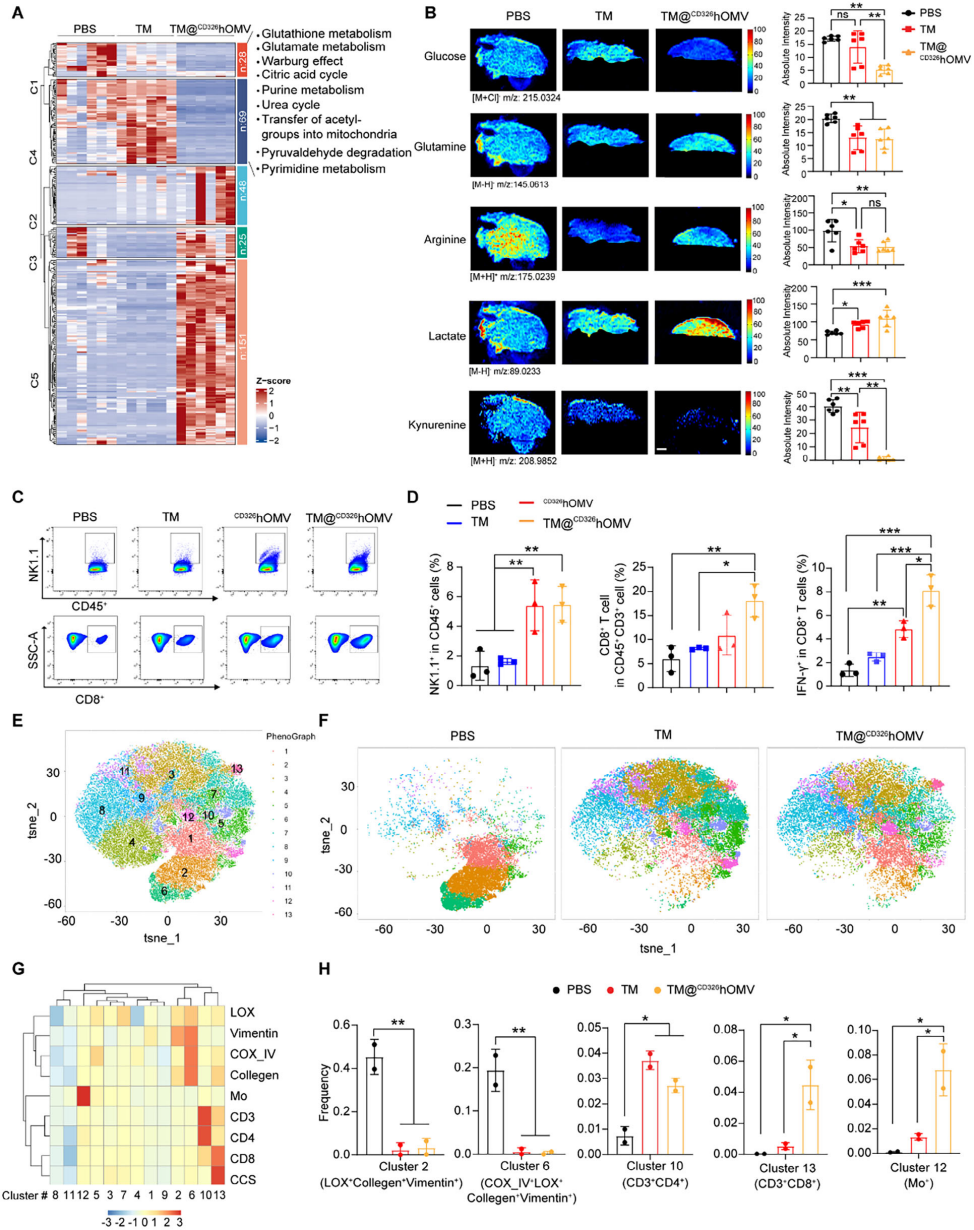
TM@^CD326^hOMV induces a tumor metabolic shift to glycolysis and enhances antitumor immunity. A) Heatmap displaying altered metabolites in tumors treated with PBS, TM, or TM@^CD326^hOMV. Altered metabolic pathways enriched by MSEA after TM@^CD326^hOMV treatment are detailed to the right. B) Representative images for the spatial distribution of glucose, glutamine, arginine, lactate, and kynurenine in tumor tissues from mice of the indicated groups (*n* = 3). The absolute intensity was quantified and shown in the right panel. The color bar denotes relative values. Data are represented as the mean ± SD (*n* = 6). C,D) Representative flow cytometry dot plots (C) and quantification (D) of immune cell ratios in tumor tissues: NK1.1^+^ cells (gated on CD45^+^ cells), CD8^+^ T cells (gated on CD45^+^CD3^+^ cells), and IFN‐γ^+^CD8^+^ T cells (gated on CD45^+^CD3^+^CD8^+^ cells). Data represent the means ± SD (*n* = 3) from three independent experiments. E,F) t‐SNE plots show cell clusters based on IMC data. Cell clusters are color coded (see rightmost subpanel). G) Clustering heatmap analysis of the indicated markers in tumors, the heatmap illustrates the median expression levels of various markers. The color bar denotes relative intensity. H) Boxplots depicting the frequency of key clusters in selected ROIs from the indicated groups. Representative data are shown as the mean ± SD (*n* = 3). *p* values were determined by one‐way ANOVA with Tukey's multiple comparisons test (B, D, and H). NS, not significant; *p < 0.05; **p < 0.01; ***p < 0.001.

Given the identified association of copper with OXPHOS and the immune‐desert phenotype, we adapted DESI‐MS and IMC to profile the tumor metabolomes and tumor‐infiltrated immune cells in TM@^CD326^hOMV‐treated 4T1 tumors. The metabolites from TM@^CD326^hOMV‐treated tumors clustered distinctly from PBS or the free TM group (Figure S10A, Supporting Information). Specifically, MSEA revealed that the TM@^CD326^hOMV‐treated tumors displayed a significant decrease in metabolites enriched in the glutamate metabolism, glutathione metabolism, the Warburg effect, and citric acid cycle pathways in comparison to the PBS group (**Figure** **5**A). Specifically, a significant decrease of glucose, glutamine, arginine, and kynurenine and a significant increase of lactate were observed in the TM@^CD326^hOMV group compared to the PBS or TM group (Figure 5B and Figure S10B (Supporting Information)), similar to the differential metabolites between high‐Cu and low‐Cu zones, indicating that TM@^CD326^hOMV treatment led to a metabolic shift toward glycolysis (Figure 5B and Figure S10B (Supporting Information)). The glycolysis‐over‐OXPHOS reprogramming of 4T1 tumor cells was further validated by analyzing the oxygen consumption rate (OCR) and extracellular acidification rate (ECAR) using a Seahorse analyzer, where a significant decrease in the basal OCR, ATP production, and maximal respiration, and a higher glycolysis capacity and reserve were found after TM@^CD326^hOMV treatment (Figure S10C–J, Supporting Information). Notably, kynurenine, a tryptophan‐derived immunosuppressive metabolite,^[^ ^25^ ^]^ was significantly reduced after TM@^CD326^hOMV treatment (Figure 5B), indicating the potential of tumor immunometabolic reshaping.^[^ ^26^ ^]^ We next resected tumor tissues and extracted RNA for RNA sequencing. Analysis of the Kyoto Encyclopedia of Genes and Genomes (KEGG) pathway revealed that most of the different expression genes (DEGs) of the TM@^CD326^hOMV versus PBS group were enriched in immune‐response‐associated and inflammatory signaling pathways, including antigen processing and presentation, T cell differentiation, and T cell receptor signaling pathway (Figure S11A, Supporting Information). Interestingly, comparing the ^CD326^hOMV versus PBS group, KEGG analysis revealed that the DEGs are enriched in immune‐response‐associated signaling pathways, including viral protein interaction with cytokines and cytokine–cytokine interaction (Figure S11B, Supporting Information), suggesting the immune modulation capability of the OMV vehicle.

Next, the tumor‐infiltrated immune cells were analyzed by flow cytometry. An increase in tumor‐infiltrating NK1.1^+^ cells (fourfold), CD8^+^ T cells (threefold), CD45^+^CD3^+^CD8^+^IFN‐γ^+^ cytotoxic T cells (sixfold), and CD4^+^ T cells (1.4‐fold) were found in TM@^CD326^hOMV‐treated tumors compared to the PBS‐treated tumors (Figure 5C,D and Figure S11C,E,F (Supporting Information)). ^CD326^hOMV without encapsulated TM also induced significant infiltration of NK cells (fourfold), CD8^+^ T cells (1.8‐fold), cytotoxic T cells (3.6‐fold), and CD4^+^ T cells (1.3‐fold) compared to the PBS group (Figure 5C,D and Figure S11F, (Supporting Information)). Additionally, we performed flow cytometry to quantify CD206^+^ macrophages (F4/80^+^CD206^+^) following TM@^CD326^hOMV treatment (Figure S11G, Supporting Information). The results showed no significant changes in CD206^+^ macrophage populations across treatment groups, suggesting that macrophages are not correlated with the T cell distribution/infiltration and the therapeutic effects (Figure S11G, Supporting Information). To elucidate the mechanism by which TM@^CD326^hOMV activates antitumor immune responses, BMDCs were treated with the PBS, TM, ^CD326^hOMV, or TM@^CD326^hOMV for 24 h, respectively. We found that TM@^CD326^hOMV induced significantly upregulation of DC maturation marker (CD80 and CD86) and antigen‐presenting molecules (MHC‐I and MHC‐II) compared to PBS and TM group (Figure S12A–D, Supporting Information). These results suggest that TM@^CD326^hOMV effectively promotes DC maturation and enhances the activation of CD8⁺ T cells, which are essential for antitumor immunity.

To further analyze the tumor subpopulations and immune microenvironment reshaped by TM@^CD326^hOMV, IMC was performed using a biomarker panel including immune‐related proteins (CD3, CD4, and CD8), Cu‐associated proteins (COX IV and CCS), and tumor metastasis/migration biomarkers (LOX, vimentin, and collagen I) (Figure 5E–H). By mapping ROIs (two 1 mm^2^ squares of each sample) with additional t‐SNE analyses, we identified a total of 13 clusters in tumors (Figure 5E). The frequency of two clusters with metastasis features, including the LOX^+^collegen^+^vimentin^+^ cluster and the LOX^+^COX IV^+^collegen^+^vimentin^+^ cluster, were both significantly decreased in the TM@^CD326^hOMV group compared to the untreated group (Figure 5G,H). Meanwhile, a significantly higher frequency of Mo^+^ (TM‐containing cell) cluster was observed in TM@^CD326^hOMV group compared to free TM or the PBS group, consistent with our previous results showing the efficient intratumoral accumulation of TM enabled by ^CD326^hOMV delivery (Figure 5G,H). Notably, a significant rise of CD3^+^CD4^+^ T cell cluster and CD3^+^CD8^+^ T cell cluster was presented in the TM@^CD326^hOMV groups compared to the untreated tumor (Figure 5G,H), demonstrating the reprogramming of the immune desert toward an immune‐inflamed phenotype. These findings suggest that TM@^CD326^hOMV inhibited tumor growth, especially the metastatic phenotype, and promoted immune cell infiltration to reshape an inflamed tumor immunotype.

### 2.6 TM@^CD326^hOMV Inhibits Lung Metastasis of Breast Cancer

**Figure 6:**
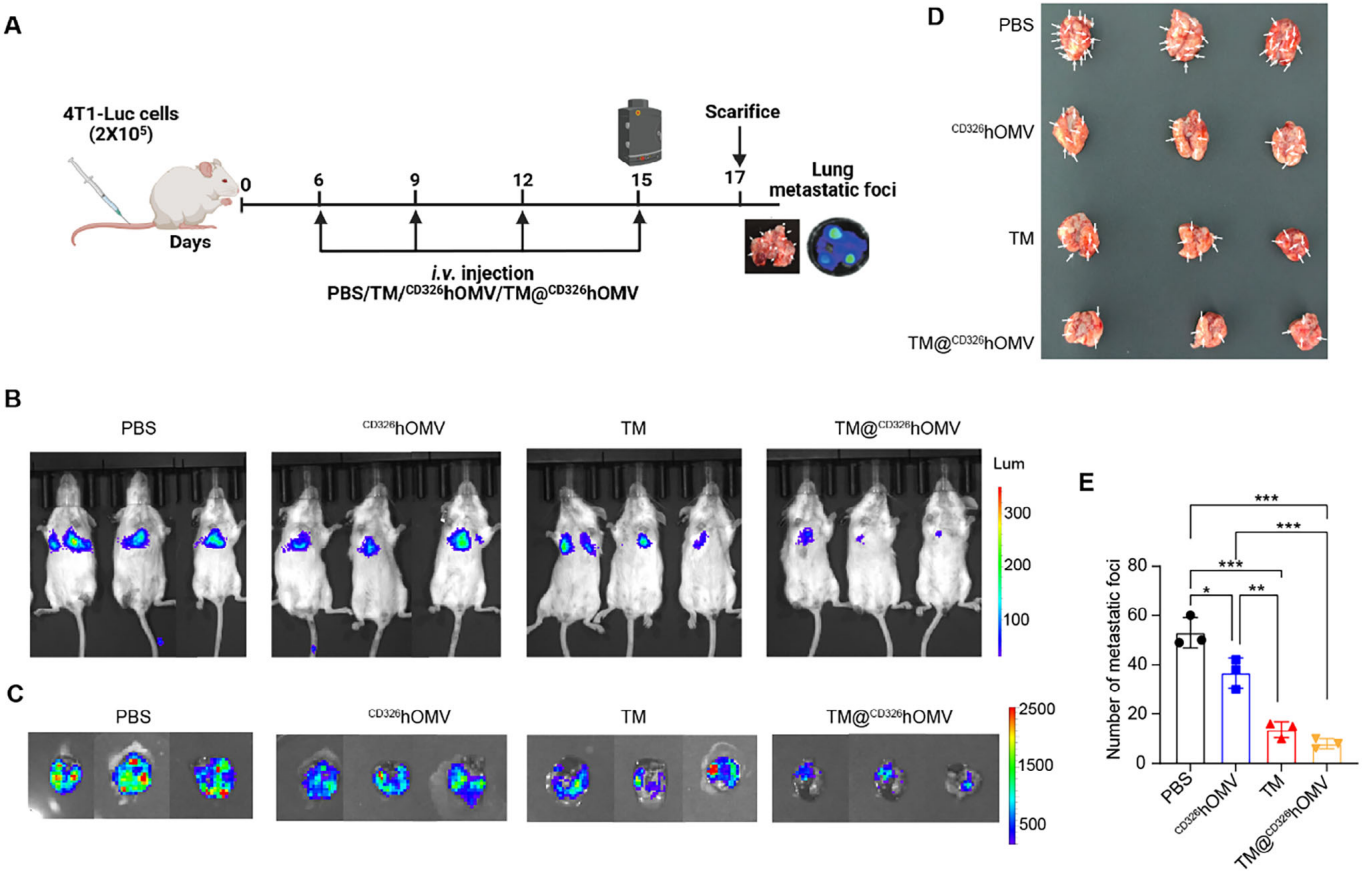
TM@^CD326^hOMV inhibited lung metastasis in 4T1‐Luc metastatic tumor models. A) 4T1‐Luciferase cells (2 × 10^5^) cells were intravenously injected into BALB/c mice. The mice were treated with PBS, ^CD326^hOMV, TM, and TM@^CD326^hOMV at equal dose of OMV (3 mg kg^−1^) and TM for 4 times. B) In vivo bioluminescence images of mice bearing 4T1‐Luc tumors treated with the indicated group on day 15. C) Representative bioluminescence images of lung metastases in each group. D) Representative photographs of lung tissue in each group. White arrows marked metastatic foci. E) Number of metastatic foci in each group (*n* = 3). Representative data are shown as the mean ± SD (*n* = 3). *p* values were determined by one‐way ANOVA with Tukey's multiple comparisons test. *p < 0.05; **p < 0.01; ***p < 0.001.

Metastasis is the leading cause of mortality in TNBC, with lung metastases occurring in 36.9% of metastatic cases.^[^ ^27^ ^]^ To assess the antimetastatic effect of TM@^CD326^hOMV, we employed the metastatic 4T1‐Luc mouse tumor model by intravenous injections of 2 × 10^5^ 4Tl‐Luc cells, which is well‐established for studying lung metastasis. After four treatment rounds at two days intervals (**Figure** **6**A), in vivo bioluminescence imaging on day 15 revealed a significant reduction in tumor burden in the TM@^CD326^hOMV‐treated group compared to controls (Figure 6B). On day 17, lungs metastases were quantified, showing a dramatic decrease in metastatic lesions. PBS‐treated mice had an average of 53 metastatic foci per lung, while ^CD326^hOMV and TM treatments reduced this to 36 and 13, respectively. Notably, TM@^CD326^hOMV treatment resulted in only 8 foci per lung (Figure 6C–E). This striking reduction—from 53 in the PBS group to 8 in TM@^CD326^hOMV‐treated mice—demonstrates its robust antimetastatic efficacy.

### 2.7 TM@^CD326^hOMV Significantly Improves the Response to αPD‐1 Blockade Therapy

**Figure 7:**
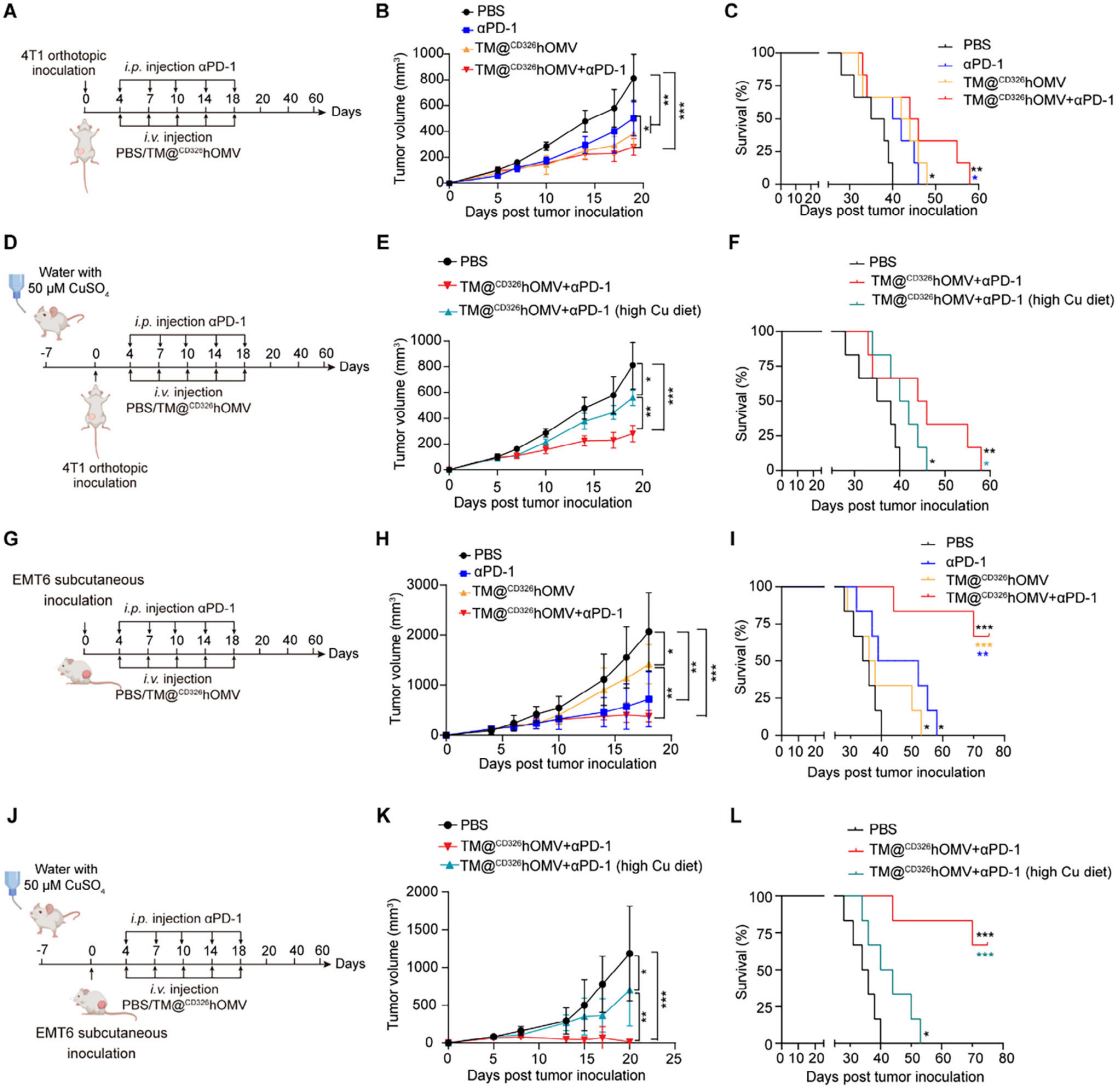
TM@^CD326^hOMV combined with αPD‐1 significantly inhibits the growth of tumors and extends mouse survival. A) Experimental design schematic for the 4T1 orthotopic model and combination and monotherapy treatments. 1 × 10^6^ 4T1 cells were injected into the mammary fat pad of BALB/c mice on day 0, followed by tail vein injection of PBS or TM@^CD326^hOMV and/or intraperitoneal injection of αPD‐1 (100 µg per mouse) on days 4, 7, 10, 14, and 18 (total of 5 times). B,C) Tumor growth curve (B) and survival data (C) for 4T1 orthotopic tumor mice given the indicated treatment with regular drinking water (*n* = 6 mice per group). D–F) Experimental design schematic (D), tumor growth curve (E), and survival data (F) for 4T1 orthotopic tumor given the indicated treatments with or without 50 µm CuSO_4_ in water (*n* = 6 mice per group). G) Schematic for the EMT‐6 subcutaneous animal model experiment. Female BALB/c mice were subcutaneously (s.c.) injected with EMT‐6 cells (3 × 10^6^ per mouse) on day 0, followed by treatment as described for panel (A). H,I) Tumor growth curves (H) and survival (I) for EMT‐6 tumor‐bearing mice receiving the indicated treatments (*n* = 6 mice per group). J–L) Scheme of experiment (J), tumor growth curves (K), and survival (L) for EMT‐6 tumor‐bearing mice receiving the indicated treatments with 50 µm CuSO_4_ in water (*n* = 6 mice per group). Data were analyzed by one‐way ANOVA (B, E, H, and K) or log‐rank (Mantel–Cox) test (C, F, I, and L) with GraphPad Prism software. NS, not significant; *p < 0.05; **p < 0.01; ***p < 0.001.

We next explored the combination potential of TM@^CD326^hOMV and immune checkpoint blockade (ICB) therapy in two murine TNBC models, including the 4T1 orthotopic model and the EMT‐6 subcutaneous model. Mice were administered with TM@^CD326^hOMV via tail vein injection, or anti‐PD‐1 (αPD‐1) (100 µg per mouse) via intraperitoneal injection, or the combination of both (**Figure** **7**A). In the 4T1 orthotopic model, combining αPD‐1 with TM@^CD326^hOMV led to a significant reduction in tumor growth compared to the αPD‐1 monotherapy (Figure 7B). Consistently, the combination therapy prolonged the median survival of 4T1‐bearing mice to 48 days, compared to 36, 41, and 42 days in the PBS, αPD‐1, TM@^CD326^hOMV groups, respectively (Figure 7C). Assuming that the copper intake would affect the therapeutic outcome, we feed mice with water containing 50 µm CuSO_4_ throughout the experiment procedure (Figure 7D). Mice receiving the combination therapy with a high‐copper diet showed significantly compromised antitumor effects compared to the regular diet group (Figure 7E), and the median survival time was significantly shortened to 42 days compared to 48 days for the regular diet‐fed mice (Figure 7F).

In the subcutaneous EMT‐6 tumor model (Figure 7G), a substantial inhibition of tumor growth was found, as 4 out of 6 mice showed complete tumor regression after treatment with the combination of αPD‐1 and TM@^CD326^hOMV (Figure 7H). Consistently, the survival of mice receiving combination therapy was significantly prolonged compared to mice in the PBS and TM@^CD326^hOMV groups, with 2 in 6 mice surviving longer than 80 days (Figure 7I). Similarly, high‐copper diet intervention of the combination therapy led to a significantly impaired antitumor effect and shortened survival time compared to the regular diet‐feed mice (Figure 7J–L). Taken together, these findings demonstrate that TM@^CD326^hOMV improved therapeutic outcomes of ICB therapy in TNBC models, and the excessive copper intake greatly undermined the therapeutic outcomes.

## 3 Discussion

Elevated copper levels have been observed in various cancers, including colorectal, gastric, breast, and gynecological tumors.^[^ ^28^ ^]^ This elevation is correlated with increased cancer severity, advanced tumor‐node‐metastasis (TNM) staging, and poorer patient outcomes, such as shorter overall survival and disease‐free survival.^[^ ^4b^ ^]^ Herein, we conducted a spatially resolved multiomics study across tumor tissues from multiple TNBC mouse models and patients, and revealed the heterogenic distribution and the propensity of copper within tumors (Figure 1), suggesting its contribution to the diverse cell‐specific metal biology in the tumor microenvironment. Notably, metabolic profiling identified differences between the 4T1 subcutaneous tumor model and patient breast cancer tissues, particularly for sodium. These differences can be attributed to the inherent limitations of the 4T1 subcutaneous tumor model, which is a nonorthotopic animal model. This model primarily reflects the intrinsic characteristics and metabolic demands of tumor cells, including features such as altered metal metabolism (e.g., elevated copper, zinc, and calcium levels). However, the 4T1 subcutaneous model does not fully replicate the unique tumor microenvironment observed in patient breast cancer tissues. In breast cancer patients, the tumor resides in a distinct microenvironment characterized by extensive infiltration of adipocytes. These adipocytes regulate fatty acid metabolism in breast cancer cells, thereby altering the tumor microenvironment and metabolic landscape.^[^ ^29^ ^]^ Interestingly, the distribution of copper, zinc, and calcium was consistent between the two models, indicating that these metals are more closely associated with intrinsic tumor cell metabolism rather than being significantly influenced by the tumor microenvironment.

High‐copper levels have been found to promote the M2 macrophage phenotype, which contributes to an immunosuppressive environment that supports tumor progression,^[^ ^30^ ^]^ suggesting the potential association of high copper with poor responses to immunotherapy. Our study, by employing spatially resolved multiomics, reveals copper‐zonation‐related tumor metabolic and immune phenotypes in breast cancers. Specifically, these high‐copper zones are associated with efficient OXPHOS, increased metastatic and proliferative markers, and immune‐desert phenotypes. Our findings expand the understanding of copper's role in driving tumor heterogeneity, suggesting that high‐copper zones are particularly pathogenic. Besides copper, our results consistently show higher levels of zinc, and sulfur in tumor tissues compared to adjacent normal tissues, regardless of the clinical subtype of breast cancer patients. Our finding aligns with previous studies on metal fingerprints,^[^ ^2b^ ^]^ which have shown that baseline metal concentrations in different organs are highly conserved, and even subtle variations in metal levels are similarly conserved.^[^ ^2^, ^31^ ^]^ These findings suggest that metals may serve as biomarkers for diagnosis and prognosis, providing valuable insights into breast cancer metabolic characteristics. In our study, TM@^CD326^hOMV treatment not only reduced copper level, but also significantly decreased iron and zinc levels (Figure S13, Supporting Information). This suggests that the observed metal changes may be attributed to shared transport mechanisms, metal‐binding proteins (e.g., metallothioneins, transferrin, or SOD1), as well as the interconnected roles of these metals in key cellular functions such as oxidative stress regulation, angiogenesis.^[^ ^12^, ^13^, ^32^ ^]^

TM is a well‐established copper chelator that binds both Cu(I) and Cu(II) ions to form stable copper─TM complexes.^[^ ^33^ ^]^ TM inhibits tumor growth through multiple mechanisms, including the suppression of angiogenesis, inhibition of tumor cell proliferation, and disruption of oxidative phosphorylation.^[^ ^34^ ^]^ Upon delivery to the tumor tissue, TM released from TM@^CD326^hOMV chelates the available copper ions. These copper─TM complexes exhibit two fates: a portion remains localized within the tumor tissue, while another portion is metabolically cleared and transported out of the tumor microenvironment. This dynamic process leads to an overall reduction in copper levels within the tumor. Traditional copper chelators such as TM and ATN224 are insufficient for treating malignancies due to their short half‐lives, off‐target copper depletion, and significant side effects, including hematological abnormalities and neurotoxicity.^[^ ^35^ ^]^ In recent years, various nanomaterials have been widely explored for tumor immunotherapy,^[^ ^36^ ^]^ offering innovative strategies to enhance therapeutic efficacy. Previous studies using copper‐chelator‐loaded polymer nanoparticles^[^ ^37^ ^]^ or mitochondria‐targeted copper‐depleting nanoparticles^[^ ^8^ ^]^ have shown inhibition of tumor growth. In this work, we developed ^CD326^hOMV for tumor‐targeted delivery of TM and demonstrated its efficient labile copper depletion efficacy in tumors and inhibitory effect on mitochondrial respiration, thus inhibiting tumor growth in various murine models. Cu‐based nanoagents have also been widely reported for tumor therapy, particularly through mechanisms such as excessive Cu‐induced Fenton reactions and cell death through cuproptosis pathway.^[^ ^6^, ^38^ ^]^ Cuproptosis is a distinct form of cell death triggered by abnormally elevated intracellular copper ion,^[^ ^9^ ^]^ which disrupts the function of iron–sulfur cluster proteins in mitochondria, leading to aberrant protein lipoylation and subsequent cell death.^[^ ^6^ ^]^ Instead of introducing copper to induce ROS or trigger cuproptosis, we employ a chelation‐based strategy to deplete copper ions in the tumor microenvironment, effectively reducing copper levels and disrupting copper‐dependent tumor growth. Beyond that, TM@^CD326^hOMV also showed additional immune reprogramming capacity and minimal impact on systemic copper homeostasis, demonstrating higher efficacy and safety.

The immunomodulatory functions of OMVs derived from probiotics, such as *E. coli* Nissle and *Akk*, are increasingly recognized for their roles in triggering innate immune signaling, inducing trained immunity, and activating T and B lymphocytes.^[^ ^22^ ^]^ In our study, we specifically designed the hybrid OMV incorporating a tumor‐targeting peptide designed for treating CD326‐overexpressed malignancies. TM@^CD326^hOMV efficiently delivered TM to the CD326^+^ tumors, and induced T cell and NK cell infiltration and cytotoxic T cell activation. This hybrid OMV platform is versatile and capable of incorporating various active pharmaceutical ingredients, including small molecules, nucleic acids, and protein drugs. The targeting peptide can be substituted with motifs such as RGD or EGFR‐targeting sequences, providing a customizable platform for precise delivery to target sites. Given the availability of OMVs from Gram‐negative bacteria, these OMV vectors can also be tailored by hybridizing with tumor‐specific or patient‐specific probiotic strains that have shown benefits in immunotherapy.^[^ ^39^ ^]^

By reversing the suppressive immune microenvironment, TM@^CD326^hOMV combined with ICB therapy improved the antitumor effect and extended survival in mice. Notably, a high‐copper diet markedly undermined the antitumor effects. According to the third National Health and Nutrition Examination Survey, the recommended daily dietary intake of copper for adults is between 1.0 and 1.6 mg.^[^ ^40^ ^]^ For breast cancer patients, it is advisable to limit copper intake by reducing consumption of copper‐rich foods, such as nuts, seeds, fish, organ meats, and dark chocolate, and avoiding using copper cookware for preparing highly acidic foods.^[^ ^41^ ^]^

Our investigation unveiled high‐copper‐zone‐related tumor pathogenesis and introduced a hybrid approach targeting tumor copper in conjunction with engineered immune‐regulatory probiotic derivatives for cancer therapy. Additionally, we emphasize the importance of considering dietary copper intake throughout tumor progression and treatment phases, especially in cancers refractory to metabolism‐targeted therapy and ICB therapy.

## 4 Experimental Section

### Medium, Reagents, and Antibodies

Dulbecco's Modified Eagle Medium (DMEM; 319‐005‐CL), RPMI 1640 medium, and fetal bovine serum (FBS; 085–150) were obtained from Wisent Bio Products (St. Bruno, Canada). Mouse GM‐CSF (Z02979‐10) and mouse IL‐4 (Z02996‐10) recombinant proteins were obtained from Genscript (Piscataway, NJ, USA). TM was purchased from Sigma‐Aldrich. Brain Heart Infusion Medium (BHI; LA0360) and radio immunoprecipitation assay lysis buffer (R0010) were purchased from Solarbio Life Science (Beijing, China). The PD‐1 antibody used for in vivo experiments was from BioXcell (BE0146, West Lebanon, NH, USA). Anti‐mouse CD16/CD32 (101302), anti‐mouse CD45‐violetFluor 450 (83090), anti‐mouse CD3‐APC‐Cy7 (17857), anti‐mouse CD8‐PE‐Cy7 (87922), anti‐mouse IFN‐γ‐APC (32793), CD3ε (E4T1B) XP mAb (78588S), CD4 (D7D2Z) mAb (25229S), CD8α (D4W2Z) XP mAb (98941), anti‐mouse MHC Class II‐violetFluor 450 (86628), EpCAM (E6V8Y) mAb (93790), HRP‐linked anti‐rabbit IgG (7074), β‐actin (13E5) mAb (4970), anti‐rabbit IgG Alexa Fluor488 conjugate (4412), and anti‐mouse IgG Alexa Fluor555 conjugate (4409) were purchased from Cell Signaling Technology (Danvers, MA, USA). Anti‐LOX (ab174316), anti‐COX IV (ab174316), anti‐collagen I (ab88147), antivimentin (ab20346), and anti‐Ki67 antibody (ab16667) were purchased from Abcam (Cambridge, MA, USA). Anti‐mouse NK1.1‐PE‐Cy5 (108716), anti‐mouse CD11c‐Brilliant Violet 605 (117334), anti‐mouse F4/80‐FITC (123108), anti‐mouse CD206‐PE (141706), anti‐mouse CD11b‐Alexa Fluor 700 (101222), anti‐mouse CD80‐PE‐Cy7 (104733), anti‐mouse CD86‐APC (105113), and anti‐mouse MHC Class I‐PE (114608) antibodies were purchased from BioLegend Inc. (San Diego, CA, USA). CCS antibody (sc‐55561) was obtained from Santa Cruz (Dallas, TX, USA). Monoclonal anti‐HA antibody (H9658) was obtained from Sigma‐Aldrich (St. Louis, MO, USA).

### Patient Tissue Samples, Animals, and Cells

Breast cancer tissues and their paired adjacent normal tissues were obtained from patients who underwent mastectomy at Peking University Cancer Hospital and Institute/Beijing Cancer Hospital. All samples were transferred to liquid nitrogen and stored at −80 °C until usage. All samples were collected with approval by the Ethics Committee of Beijing Cancer Hospital (No. 2024KT18), and informed consent was obtained from all patients. Clinical information of the patients was shown in Table 1.

Female BALB/c mice (6–8 weeks old) were purchased from SPF Biotechnology Co. Ltd. (Beijing, China). OT‐1 mice were kindly gifted by Dr. Yating Wang (School of Basic Medicine Science, Tsinghua University, Beijing, China). Mice were housed in a room with a temperature of 20–25 °C and a humidity of 30–70%. Feed and water were available ad libitum. Artificial light was provided in a 12 h light/12 h dark cycle. All animal protocols were approved by the Institutional Animal Care and Use Committee of the National Center for Nanoscience and Technology. The assigned approval/accreditation number was NCNST‐LX‐2203‐19. The 4T1, MDA‐MB‐231, MEF, A549, Hela, and MCF‐7 cell lines were obtained from the American Type Culture Collection [(ATCC), Manassas, VA, USA]. EMT‐6 cells were generously provided by Feng Shao at the National Institute of Biological Sciences, Beijing. 4T1, MDA‐MB‐231, MEF, A549, HeLa, and MCF‐7 cells were cultured in DMEM containing 10% FBS, 1% penicillin, and streptomycin in an incubator at 37 °C with 5% CO_2_. EMT‐6 cells were cultured in RPMI‐1640 medium supplemented with 10% FBS. All cell lines were tested mycoplasma‐free.

### Plasmid Construction, Bacterial Strain, and Growth

The gene encoding ClyA–HA–CD326 was synthesized and cloned into the expression plasmid pGEX‐6P‐1 (Genewiz, Suzhou, China). The *E. coli* strain *BL21* (DE3) transformed with pGEX‐6P–ClyA–HA–CD326 was grown at 37 °C in LB medium with shaking at 180 rpm. After the culture had reached an optical density at 600 nm of 0.6, protein expression was induced by the addition of 0.1 mm isopropyl β‐d‐1‐thiogalactopyranoside (0.1 mm) at 16 °C for 16 h. Ampicillin (100 µg mL^−1^) was added when appropriate. Gram‐negative anaerobic bacteria *Akk* (ATCC BAA‐835) was purchased from the American Type Culture Collection (Manassas, VA, USA) and cultured in BHI at 37 °C in a 90% N_2_, 5% H_2_, and 5% CO_2_ anaerobic atmosphere.

### Isolation and Purification of OMVs

To isolate OMVs from cultured bacterial supernatants, *E. coli* was cultured as described above and removed by centrifugation at 6000 × *g* for 10 min at 4 °C. *Akk* was removed by centrifugation at 8000 × *g* for 15 min at 4 °C. The resulting supernatant (300 mL) was filtered through a 0.45 µm EPS filter (R8SA47939, Millipore), and then concentrated to 30 mL using a 100K ultrafiltration tube (UFC710008, Millipore). The concentrated solution was further filtered through a 0.22 µm EPS membrane (R3EA06699, Millipore). OMVs were collected from the filtrate by ultracentrifugation at 150 000 × *g* for 2 h at 4 °C, then resuspended in 400 µL PBS and finally passed through a 0.22 µm filter to remove cell debris. The filtrated OMV was stored at −80 °C until use. The total protein concentration of OMVs was evaluated using the bicinchoninic acid assay and the results of which were defined as the OMV concentrations.

### Preparation and Characterization of TM@^CD326^hOMV

^CD326^hOMV was prepared by fusing the ^CD326^ *BL21*‐OMV with the *Akk*‐OMV at a protein mass ratio of 1:2. The mixture was emulsified by ultrasonication (50 W) on ice for 5 min, followed by being repeatedly extruded through 400 and 200 nm polycarbonate film (Whatman), respectively. TM was loaded into ^CD326^hOMV by electroporation (750 V, 50 µF, and 200 Ω) using the Gene Pulser Xcell Electroporation System (Bio‐Rad, CA, USA). The loading efficiency and the cumulative release of TM were calculated based on the specific absorbance at 468 nm. The morphology and size distribution of TM@^CD326^hOMV were characterized using TEM (HT7700 electron UK), respectively. Briefly, resuspended vesicles (10 µL) were dropped onto copper grids that had been exposed to UV light to reduce static electricity. After drying for 20 min, OMVs were negatively stained with 2% uranyl acetate for 6 min. The dried grids were viewed by TEM at 80 kV. For the size, ζ potential, and polydispersity index of OMVs, 1 mL of PBS containing vesicles at 50 µg mL^−1^ total protein was analyzed using a Zetasizer Nano ZS dynamic light scattering instrument (Malvern Instruments, UK).

### Sample Preparation for LA‐ICP‐MS, DESI‐MSI, IMC, and Histological Examination

Tumors were collected, immediately shock‐frozen in liquid nitrogen, and kept at −80 °C until analysis. For LA‐ICP‐MS measurements, tumors were cryosectioned into slices of 10 µm thickness using a cryomicrotome (Leica Biosystems, Wetzlar, Germany). The cryosections were placed on glass slides, air‐dried, and kept at room temperature until analysis. For DESI‐MS measurements, tumors were cryosectioned into slices of 10 µm thickness and store at −80 °C until analysis. For IMC measurements, tumors were cryosectioned into slices of 6 µm thickness and stored at −80 °C until analysis. A consecutive cryoslice of 10 µm of the respective organ was stained with hematoxylin–eosin (H&E) using standard protocols. Other tissues were paraffin‐embedded, sliced, and H&E‐stained using routine histological methods.

### LA‐ICP‐MS Analysis

The LA‐ICP‐MS measurements were performed using a NWR213 laser ablation (LA) system (ESI, Fremont, CA, USA) coupled to a quadrupole ICP‐MS instrument (PerkinElmer, NexION 300D, Norwalk, CT, USA). It allowed the simultaneous measurement of the mass range of *m*/*z* from 6 to 238. Daily performance and fine‐tuning of the ICP‐MS were performed with a NIST612 in line scan mode to achieve a maximum signal intensity of ^115^In^+^ and ^238^U^+^ and a ratio of ^238^U^16^O^+^/^238^U^+^ (<3%). The parameters used in the measurements were: Rf power input: 1300 W, argon plasma gas flow rate: 15 L min^−1^, nebulizer gas flow rate: 0.95 L min^−1^, helium carrier gas flow rate: 1 L min^−1^, dwell time: 13 ms, mass resolution: max. 500 *m*/Δ*m*, scanning mode: peak hopping, laser spot size: 60 µm, scan speed: 70 µm s^−1^, ablation mode: line scan, repetition frequency: 20 Hz, laser fluence: 1.6 J cm^−2^. The images of LA‐ICP‐MS were reconstructed using MATLAB software, and then processed with Advanced Normalization Tools (UPENN, USA), corrected for intensity nonuniformity caused by surface coil reception using the N4 algorithm. ROI were selected and the average signal values were calculated by MATLAB software.

### DESI‐MSI Analysis

DESI‐MSI analysis was performed in both positive and negative ion modes at *m*/*z* 10–1000 on a Q‐Exactive orbitrap mass spectrometer (Thermo Scientific). The spray solvent was a mixture of acetonitrile and water (8:2, v/v) at a flow rate of 5 µL min^−1^. The spray voltages were set at −5000 V in negative‐ion mode and 5000 V in positive‐ion mode. The extracting gas flow was 20 L min^−1^, and the capillary temperature was 350 °C. Imaging analysis was performed by continuously scanning the tissue section in the *x*‐ and *y*‐directions at a constant rate of 100 µm s^−1^. MassImager software was used for background subtraction, image reconstruction, and the calculation of average metabolite expressions in the region of interest. The separated sample dataset matrixes were then imported into the Markerview software for peak picking, peak alignment, and isotope removing (process spectra options: the mass tolerance was 0.01 Da). PCA and OPLS‐DA were performed to compare the differences in metabolic substance between groups. SIMCA‐P 14.0 software was used for multivariate statistical data analyses. Pathway analyses were applied using MSEA using MetaboAnalyst 6.0.

### IMC Analysis

Purified antibodies lacking carrier protein were conjugated with metal reporters by using a Maxpar X8 antibody labeling kit (Fluidigm). Tumor sections were fixed using 4% paraformaldehyde for 30 min at 4 °C. After fixation, tissue sections were washed in Dulbecco's phosphate‐buffered saline (ThermoFisher Scientific) containing 0.5% bovine serum albumin and 0.05% Tween, rehydrated in additive‐free Dulbecco's phosphate‐buffered saline, followed by washing and blocking with 3% bovine serum albumin in PBS. Tissue sections were then stained with a cocktail of metal‐conjugated antibodies overnight at 4 °C, washed, and incubated with 125 nm Cell‐ID Intercalator‐Ir for 30 min at room temperature. After 3 times washing, tissue sections were dipped in Milli‐Q water (Merck Millipore) for 5 min and dried for 20 min at room temperature. Data were acquired using a Hyperion imaging mass cytometer (Fluidigm, CA, USA) at a resolution of 1 µm, with settings aligned to company guidelines. The ablation frequency was 200 Hz, and the energy was 3 dB. Regions of interest were acquired at a size of 1 × 1 mm^2^.

Raw IMC data were analyzed with a pixel‐classification‐based multiplexed image segmentation pipeline (https://github.com/BodenmillerGroup/ImcSegmentationPipeline). Image preprocessing and supervised pixel classification were performed, and then CellProfiler was used for image segmentation and quantification of single cells. Raw data transformed into cell masks and TIFF files were used in histoCAT, where both single cell and neighborhood analysis could be performed or extracted as single‐cell feature. Single‐cell measurements were then extracted from all available channels using the mean pixel values for each segmented cell. The data were normalized by using 99th‐percentile for t‐SNE (parameters: initial dimensions, 110; perplexity, 30; theta, 0.5). PhenoGraph was used for unsupervised clustering, and the single‐cell protein expression data were arcsine transformed. These clusters were aggregated and classified into cell subtypes based on marker expression. Here, Mo (indicating TM), and metal‐labeled antibodies against CD4, CD8, CD3, collagen I, CCS, LOX, COX IV, and vimentin were detected for the clustering.

### Measurement of OCR and Extracellular Media Acidification Rate

OCR and ECAR of cells were measured with the Seahorse XFe96 Extracellular Flux Analyzer (Seahorse Bioscience) according to the manufacturer's instructions. In brief, 4T1 cells (5000 cells per well) were cultured in FN‐precoated XFe96 well plates with medium, then were treated with 2.6 µg mL^−1^ TM and 10 µg mL^−1^ TM@^CD326^hOMV for 16 h. Before OCR measurement, cell medium was replaced by XF assay medium (XF Base Medium supplemented with 2.5 mm glucose, 1 mm pyruvate, 2 mm l‐glutamine and adjusted to pH 7.4). Before ECAR measurement, cell medium was replaced by XF assay medium (XF Base Medium supplemented with 2 mm l‐glutamine and adjusted to pH 7.4). Cells were then maintained in 175 µL per well of XF assay medium at 37 °C in an incubator without supplied CO_2_ for 1 h. During the incubation time, 1 µm oligomycin, 0.5 µm FCCP, 1 µm rotenone, and 1 µm antimycin A in XF assay medium were loaded into the injection ports in the XFe96 sensor cartridge for the OCR test. For the ECAR test, 10 mm glucose, 1 ²m oligomycin, and 50 mm 2‐DG in XF assay medium were loaded. The assay procedure was set as “Mix‐03:00, Wait‐00:00, Measurement‐03:00” for 4 cycles. After measurement, cells were digested with trypsin and counted for cell number. OCR and ECAR of cells were normalized to cell numbers. Datasets were analyzed by Wave software (Agilent Technologies). The experiment was performed with at least five replicates per condition.

### Copper Depleting Dynamics of TM@^CD326^hOMV In Vitro and In Vivo

The labile copper level was measured by CCL‐1 luminescence imaging. For in vitro copper depletion, 4T1‐Luc cells were seeded in 48‐well plates (20 000 per well), then treated with TM or TM@^CD326^hOMV (50 µm) and incubated for 0, 3, 6, 12, and 24 h. After washing 3 times with PBS, 100 µL of CCL‐1 in D‐PBS (50 µm) was added to each well and immediately imaged by IVIS Spectrum (PerkinElmer). For in vivo copper depletion, 4T1‐luc tumor‐bearing mice were administrated with TM (25 µg per mouse) or TM@^CD326^hOMV (i.v., 60 µg protein containing 25 µg TM per mouse) and imaged at 3, 6, 12, 24, and 48 h postinjection. For each measurement, the mice were intraperitoneally injected with freshly prepared CCL‐1 (6 mg kg^−1^ in a D‐PBS solution) 30 min prior to the IVIS imaging for luminescence signal.

### RNA Sequencing and Analysis

Briefly, total RNA isolation, sample quality check, and library preparation were performed by BGI (Wuhan, China). The data analysis was performed on Dr. Tom Multiomics Data Mining System (https://biosys.bgi.com). Differential gene analysis was performed using DESeq2 with a *Q* value ≤ 0.05. KEGG enrichment analysis of annotated different expression genes were performed by Phyper based on a hypergeometric test. The significant levels of pathways were corrected by a *Q* value with a rigorous threshold (*Q* value ≤ 0.05). The RNA‐seq data were deposited in the NCBI Sequence Read Archive database, with accession number (PRJNA1113789).

### Statistical Analysis

Data were presented as the mean ± standard deviation (SD). At least three independent experiments were performed for each in vitro study. Data were analyzed by a two‐tailed unpaired *t* test or one‐way ANOVA with Tukey's multiple comparisons test for comparison of two groups, and a two‐way ANOVA followed by Bonferroni posttest analysis for comparison of more than two groups of data. The mouse survival was compared using the log‐rank (Mantel–Cox) test. Heatmap, PCA analysis, correlation network, and volcano plot were plotted by https://www.bioinformatics.com.cn, an online platform for data analysis and visualization. Origin Pro 8.5.1, GraphPad Prism 5, FlowJo V10, and ImageJ v1.8.0 were used to analyze the acquired data. Statistical significance was set as follows: *p < 0.05; **p < 0.01; ***p < 0.001; NS, not significant.

## Conflict of Interest

The authors declare no conflict of interest.

## Author Contributions

L.C., S.M., and H.W. contributed equally to this work. Conceptualization: L.C., C.Z., J.C., M.W., and M.Z. Methodology: L.C., S.M., H.W., L.Z., Y.Y., G.L., B.L., J.S., B.W., Y.L., Y.P., W.W., and M.W. Patient sample acquisition: L.C., H.W., and C.Z. Visualization: L.C., Y.P., and M.Z. Supervision: J.C., M.W., and M.Z. Writing—original draft: L.C., S.M., and M.Z. Writing—review and editing: L.C. and M.Z.

## Supporting information



Supporting Information

## Data Availability

The data that support the findings of this study are available from the corresponding author upon reasonable request.
