# Supplementary material for: Zonated Copper‐Driven Breast Cancer Progression Countered by a Copper‐Depleting Nanoagent for Immune and Metabolic Reprogramming
Source: Adv Sci (Weinh). 2025 Apr 24;12(20):2412434. doi: 10.1002/advs.202412434 (PMC12120698; doi:10.1002/advs.202412434)
Supplement: Supplementary file 1 — Supporting Information [file ADVS-12-2412434-s001.docx]

**Supporting Information for**

**Zonated copper-driven breast cancer progression countered by a copper-depleting nanoagent for immune and metabolic reprogramming**

*Lin Chen^†^, Saibo Ma^†^, Hao Wu^†^, Lingna Zheng, Yunpeng Yi, Guangnian Liu, Baoyi Li, Jiayi Sun, Yang Du, Bing Wang, Yike Liu, Cheng Zhang, Jing Chang*, Yuheng Pang, Wenjing Wang, Meng Wang*, Motao Zhu**

^†^These authors contributed equally to this work

^*^Correspondence:

Meng Wang: [wangmeng@ihep.ac.cn](mailto:wangmeng@ihep.ac.cn);

Jing Chang: [jingchouc@163.com](mailto:jingchouc@163.com)

Motao Zhu: [zhumt@nanoctr.cn](mailto:zhumt@nanoctr.cn)

**Supporting Materials and methods**

**Fluorescence resonance energy transfer (FRET)**

For the FRET assay, *Akk*-OMV and ^CD326^*BL21*-OMV were labeled with DiO (as the energy donor) and DiL (as an acceptor), respectively. ^CD326^Hybrid-OMV was prepared by hybridizing DiO-*Akk*-OMV and DiL-^CD326^*BL21*-OMV using ultrasound and extrusion. The fluorescence spectra of DiO-*Akk*-OMV, DiL-^CD326^*BL21*-OMV, ^CD326^Hybrid-OMV and the physical mixture of the two OMVs were measured on Cary Eclipse Fluorescence Spectrophotometer (Agilent Technologies). The emission spectra were scanned from 500 nm to 700 nm with an excitation wavelength of 480 nm at room temperature in PBS buffer. The background signal of the PBS buffer was subtracted from the FRET signal in all measurements.

**Western blotting analysis**

The protein amounts were qualified using a BCA protein assay kit and separated by 12% sodium dodecyl sulfate-polyacrylamide gel electrophoresis and transferred to 0.45 μm PVDF membranes. The membranes were blocked with 5% nonfat milk in TBS with 0.1% Tween 20 (TBST) for 1 h at room temperature, and probed with primary antibody for 16 h at 4 °C followed by secondary antibody at room temperature for 2 h. Immunoreactive proteins were visualized using the Invitrogen Novex ECL Chemiluminescent Substrate Reagent Kit.

**Confocal microscopy observation of cellular uptake**

To assess the interaction of TM@hOMV or TM@^CD326^hOMV with the cell membrane, 4T1 and HeLa cells were seeded onto 35 mm confocal microscopy dishes in DMEM medium containing 10% FBS. Cells were incubated with FITC-labeled TM@^CD326^hOMV or TM@hOMV (2 μg/mL)­ at 4 °C for 1 h. Cell nuclei (blue) were stained with Hoechst 33342, and the cells were observed by a confocal microscope (Zeiss LSM710, Germany). To observe the cellular uptake, 4T1 cells were incubated with FITC-labeled TM@^CD326^hOMV or TM@hOMV at 37 °C for 1 and 3 h, respectively. The cells were harvested for flow cytometry analysis.

***In vivo* biodistribution of TM@^CD326^hOMV**

To evaluate the biodistribution of TM@^CD326^hOMV in vivo, TM@^CD326^hOMV was labeled with the fluorescent dye Cy5.5 via ultrasonic emulsification. Cy5.5-labeled TM@^CD326^hOMV (60 µg protein per mouse) were then injected into 4T1 tumor-bearing mice via the tail vein. At 1, 5, 6, 12, 24, and 48 h post-injection, the mice were imaged. At 24 h post-injection, the major organs and tumors of the mice were harvested for *ex vivo* imaging. The fluorescent signal was determined by biphotonic imaging using an IVIS spectrum system (PerkinElmer, USA).

**DC maturation and antigen presentation *in vitro***

To investigate the effects of TM@^CD326^hOMV on DC maturation and antigen presentation *in vitro*, BMDCs were extracted from the bone marrow of healthy C57BL/6J mice and cultured with interleukin-4 (IL-4), granulocyte-macrophage colony-stimulating factor (GM-CSF). On day 5, immature BMDCs were treated with PBS, TM, ^CD326^hOMV and TM@^CD326^hOMV. Following 24 h of treatment, BMDCs were stained with anti-mouse CD11c-BV605, anti-mouse CD80-PE/Cy7, anti-mouse CD86-APC antibodies, anti-mouse MHC-I-PE, and anti-mouse MHC-II-violetFluor™ 450 antibodies to assess their maturation and antigen presentation via flow cytometry.

**Experimental design for OVA^257-264^-specific CD8^+^ T cell activation**

OVA^257-264^-specific CD8^+^ T cells were isolated from the spleens of OT-1 mice using the Dynabeads Untouched Mouse CD8 Cells Kit (11417D), following the manufacturer's instructions. BMDCs were isolated from C57BL/6J mice and co-cultured with OVA^257-264^-specific CD8^+^ T cells in the presence of OVA^257-264^ peptide with ^CD326^hOMV or ^CD326^BL21-OMV. After 24 h of co-culture, IFN-γ production in the supernatant was quantified using an ELISA assay.

**Tumor model establishment and therapeutic efficacy evaluation**

To establish a 4T1 subcutaneous tumor model, 6-8 weeks old female BALB/c mice were subcutaneously administered with 4T1 tumor cells (2 x 10^6^ per mouse) on the right flank. To establish an EMT-6 subcutaneous tumor model, 6-8 weeks old female BALB/c mice were subcutaneously administered with EMT-6 tumor cells (3 x 10^6^ per mouse) on the right flank. To establish an MCF-7 subcutaneous tumor model, 6 weeks old female BALB/c-nu mice were subcutaneously administered with MCF-7 tumor cells (8 x 10^6^ per mouse) on the right flank. For animal models used in labile copper bioluminescence imaging experiments, 2 x 10^6^ 4T1-Luc cells were inoculated subcutaneously into the right dorsal flank of the mouse. For orthotopic model, 1 x 10^6^ 4T1 cells were inoculated into the three and fourth mammary fat pads of female BALB/c mice.

To test the effects of TM@^CD326^hOMV in anti-tumor therapy, the tumor bearing mice were treated with (1) PBS, (2) ^CD326^hOMV (60 µg protein per mouse), (3) TM (25 µg per mouse), and (4) TM@^CD326^hOMV (60 µg protein containing 25 µg TM per mouse). The tail vein injections were started on day 4 after tumor inoculation. The mice received a total of 5 treatments of TM@^CD326^hOMV on day 4, 7, 10, 14, and 18. Tumor volume was calculated according to the following formula: tumor volume = length x 1/2 width^2^. The mice were euthanized on day 20. The plasma was collected to analyze the metal content and the tumors were collected, weighed, and digested into single-cell suspensions to analyze the infiltrating immune cells by flow cytometry.

To test the effect of TM@^CD326^hOMV on lung metastasis, 2 x 10^5^ of 4T1-Luc cells were intravenously injected to establish lung metastatic tumor models. On day 6, 9,12 and 15, the mice were intravenously treated with (1) PBS, (2) ^CD326^hOMV (60 µg protein per mouse), (3) TM (25 µg per mouse), and (4) TM@^CD326^hOMV (60 µg protein containing 25 µg TM per mouse). On day 15, bioluminescence imaging was performed using an IVIS system 10 min after intraperitoneal injection of D-luciferin potassium salt (150 mg/kg). On day 17, the BALB/c mice received an intraperitoneal injection of D-luciferin potassium salt and were sacrificed 5 min later. The lungs were harvested and imaged by IVIS system. Metastatic lesions were quantified, and representative photographs and bioluminescence images were acquired for each experimental group.

To test the effect of TM@^CD326^hOMV on improving ICB therapy, tumor-bearing mice were administered TM@^CD326^hOMV via tail vein injection, αPD-1 (100 µg per mouse) via intraperitoneal injection, or the combination of both, on days 4, 7, 10, 14, and 18, for a total of five times. To evaluate the impact of copper intake, drinking water was supplemented with 50 µM CuSO_4_ in the high copper diet group.

**Analysis of different cell populations and phenotypes in tumors**

The tumors were harvested and sliced into tiny pieces in cold PBS. The fragments of tumor tissue were mechanically broken up and then incubated in 5 mL of digesting solution (RPMI 1640 containing 1.5% FBS, 0.5 mg/mL collagenase IV, 0.5 mg/mL collagenase I and 0.04 mg/mL DNase I) at 37 °C for 60 min under shaking (200 rpm), followed by vertexing for 1 min before passing through a 70 µm cell strainer. Single-cell suspensions were recovered in PBS and analyzed using flow cytometry. To minimize nonspecific Fc receptor binding, cells were blocked with anti-mouse CD16/CD32 for 30 min. To analyze tumor infiltrated T cell populations, cells were stained with CD45-violetFluor™ 450, CD3-APC-Cy7, CD8-PE-Cy7, CD4-Percp-Cy5, and IFN-γ-APC. To analyze NK cells in tumors, cells were stained with CD45-violetFluor™ 450, and NK1.1-PE-Cy5. To analyze macrophage in tumors, cells were stained with CD45-violetFluor™ 450, CD11b-AF7000, F4/80-FITC, and CD206-PE. Fluorescent signals were assessed using an Attune NxT flow cytometer (Thermo, USA). FlowJo software was used to analyze the results.

**Inductively coupled plasma mass spectroscopy (ICP-MS)**

For tumor tissue, approx. 100 mg of tumor tissue were dried in a freeze dryer for 24 h, and then weighed. Dried tumor tissue was digested using nitric acid in a sealed high-pressure digestion vessel and incubated on a heating plate at 100 °C until the solution became yellow and transparent. The temperature was increased to 140 °C to drive off the acid. After the liquid in the vessel evaporated to around 100 μL, hydrogen peroxide was added until the solution clears. The digested sample was diluted with 2% nitric acid to 20 mL and subjected to ICP-MS analysis (PerkinElmer, NexION 300D, Norwalk, USA). Metal levels were normalized to the tumor dried weight. For cells and mouse plasma metal concentrations, cells (100 μg protein per sample) or 50 μL plasma were incubated with 200 μL HNO_3_ at 100 °C for 4 h. All samples were made up to a total volume of 5 mL with 2% nitric acid and analyzed using ICP-mass spectroscopy (PerkinElmer, NexION 300D, Norwalk, USA). The metal standards prepared with 2% nitric acid were used to calibrate the standard curve.

**Supporting Figures**


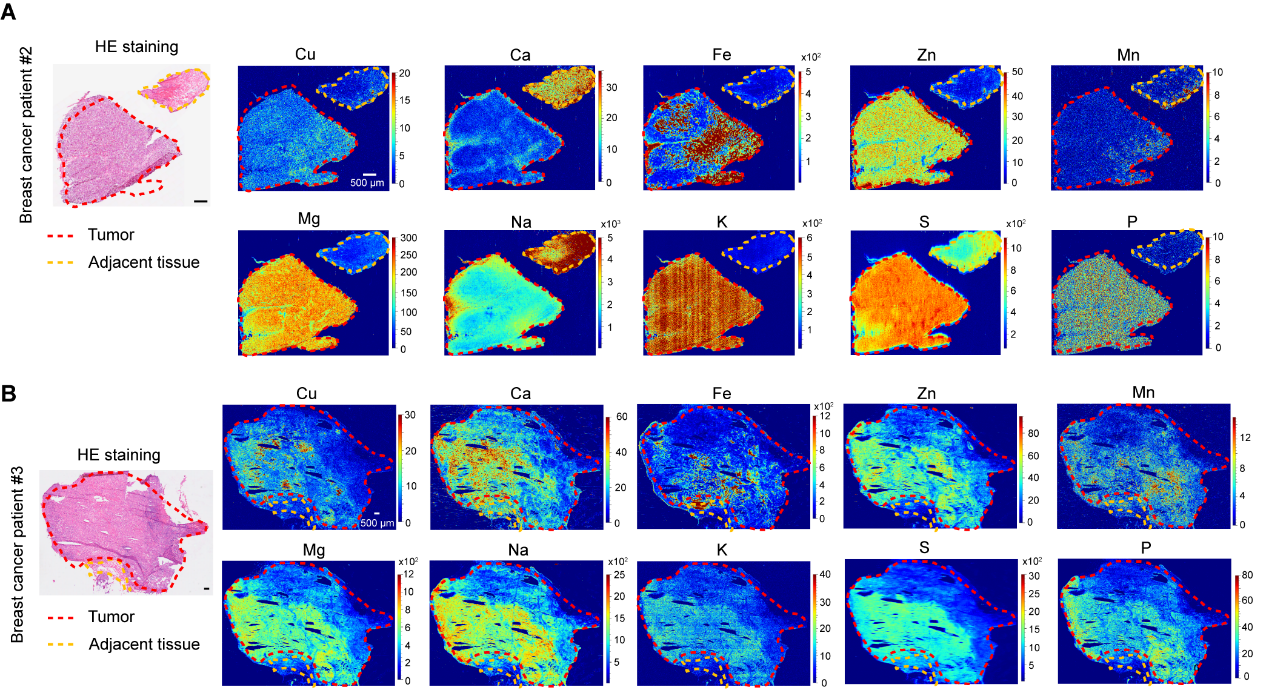


**Figure S1.** LA-ICP-MS images of the metallome of tumor and adjacent tissues in breast cancer patients. Representative MS images are shown for breast cancer patient (A and B). The hydrogel-embedded sections were analyzed for copper (^63^Cu), calcium (^44^Ca), zinc (^64^Zn), iron (^56^Fe), manganese (^55^Mn), magnesium (^24^Mg), sodium (^23^Na), sulfur (^34^S), potassium (^39^K), and phosphorus (^31^P). Scale bar: 500 μm.


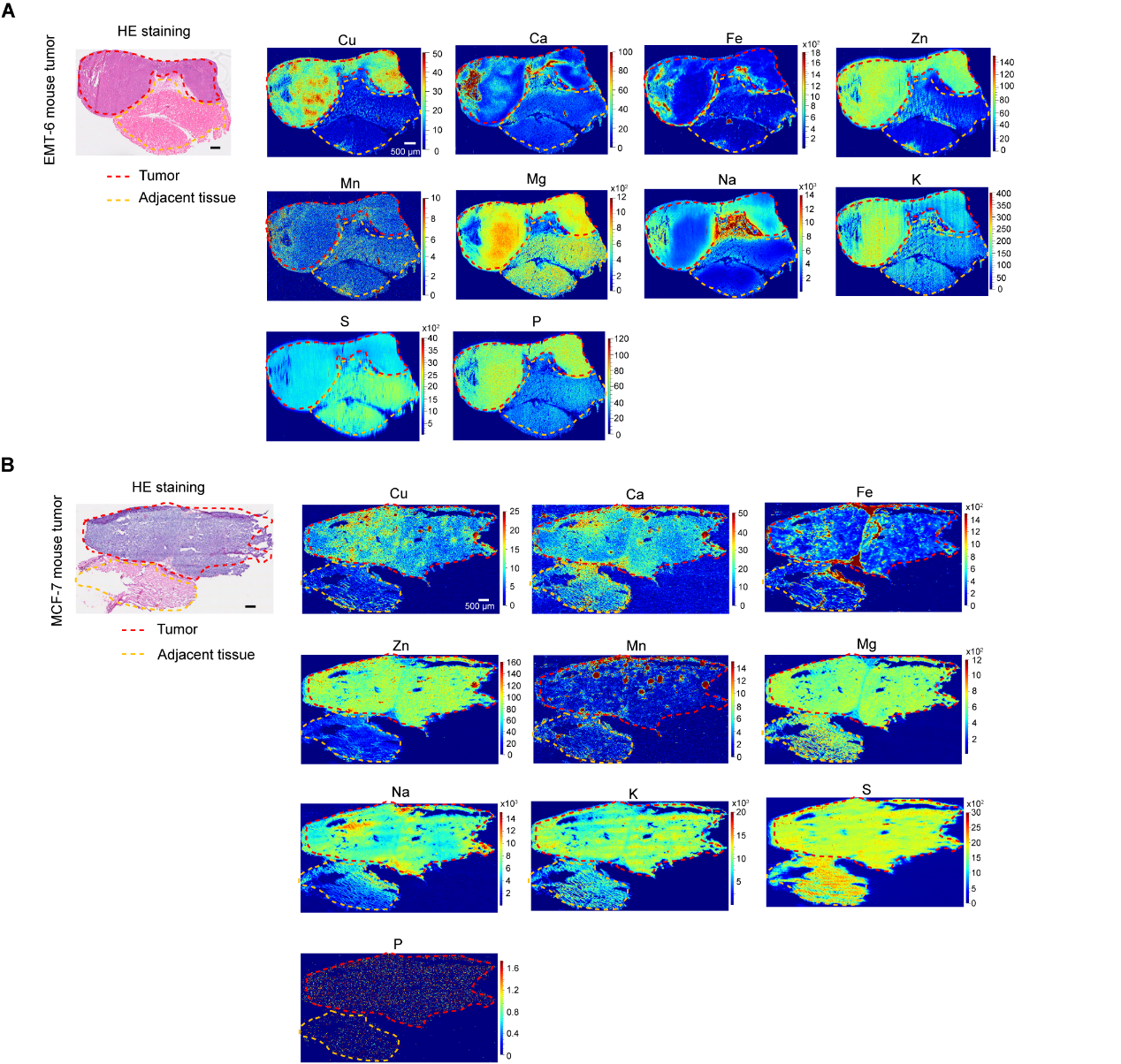


**Figure S2.** Spatial metallomics of tumor and adjacent tissues from mouse models. Representative spatial metal distribution in tumors of EMT-6 (A), and MCF-7 (B) mouse models. The hydrogel-embedded tumor sections were analyzed for copper (^63^Cu), calcium (^44^Ca), zinc (^64^Zn), iron (^56^Fe), manganese (^55^Mn), magnesium (^24^Mg), sodium (^23^Na), sulfur (^34^S), potassium (^39^K), and phosphorus (^31^P). Scale bar: 500 μm.


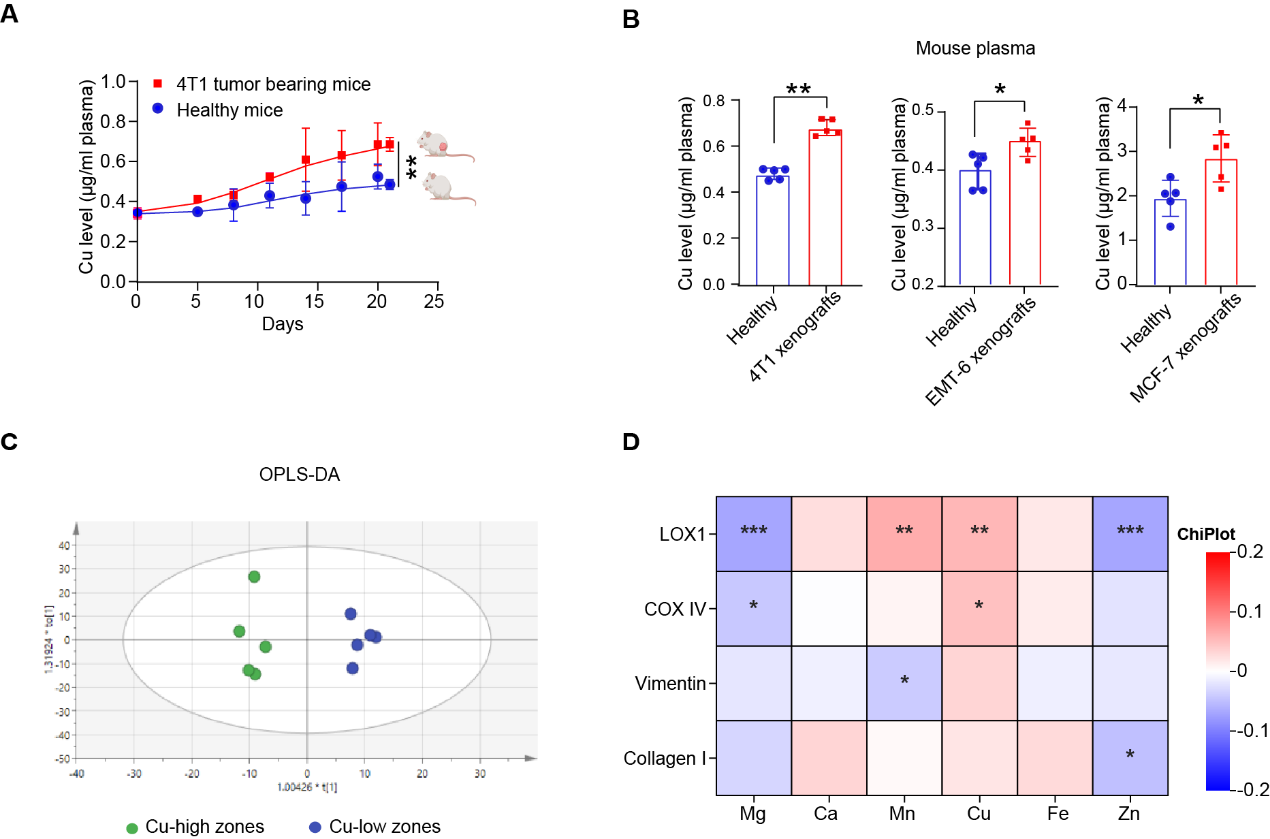


**Figure S3.** Increased copper levels in the plasma of tumor-bearing mice compared to healthy individual. A,B) ICP-MS analyses of plasma copper levels in 4T1 tumor-bearing mice and healthy mice starting from the day of tumor inoculation (A) and at the day 20 post 4T1, EMT-6, and MCF-7 tumor-inoculation (B). Data are represented as the mean ± SD (n = 5). C) OPLS-DA score plot of mice tumor tissue metabolites from five ROI regions in Cu high zones and Cu low zones in three independent sample. D) The heatmap illustrates the correlation between metals and ECM components.


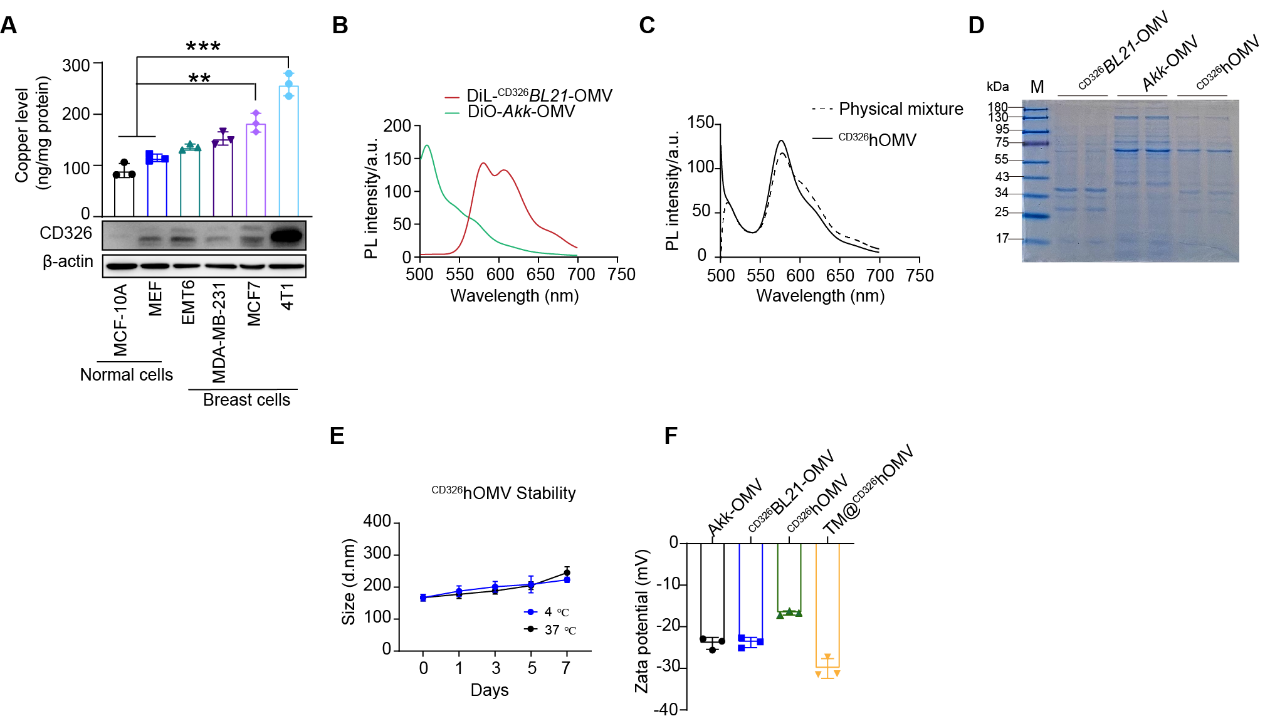


**Figure S4.** Characterization of TM@^CD326^hOMV. A) ICP-MS measurements of the Copper content (upper panel) in multiple normal and breast cancer cell lines. Immunoblotting analyses of the corresponding CD326 protein levels are shown (lower panel). β-actin serves as an internal control. Data are represented as the mean ± SD (n = 3). B,C) FRET assay using nanovesicles labeled with lipophilic fluorescent dye. *Akk*-OMV@DiD and ^CD326^*BL21*-OMV@Dil were excited at 480 nm, and the emission was collected between 500-700 nm. ^CD326^hybrid-OMV has an increased emission peak at 570 nm compared the physical mixture group. D) Coomassie brilliant blue staining of proteins extracted from *Akk*-OMV, ^CD326^*BL21*-OMV, and ^CD326^hOMV, respectively. E) DLS analysis of ^CD326^hOMV incubated at 4°C or 37°C for the indicated days. Data are representative of three independent experiments. F) Zeta potentials of *Akk*-OMV, ^CD326^*BL21*-OMV, ^CD326^hOMV, and TM@^CD326^hOMV. Quantitative data are represented as the mean ± SD (n = 3). P values were determined by one-way ANOVA with Tukey’s multiple comparisons test in A. **P < 0.01; ***P < 0.001.


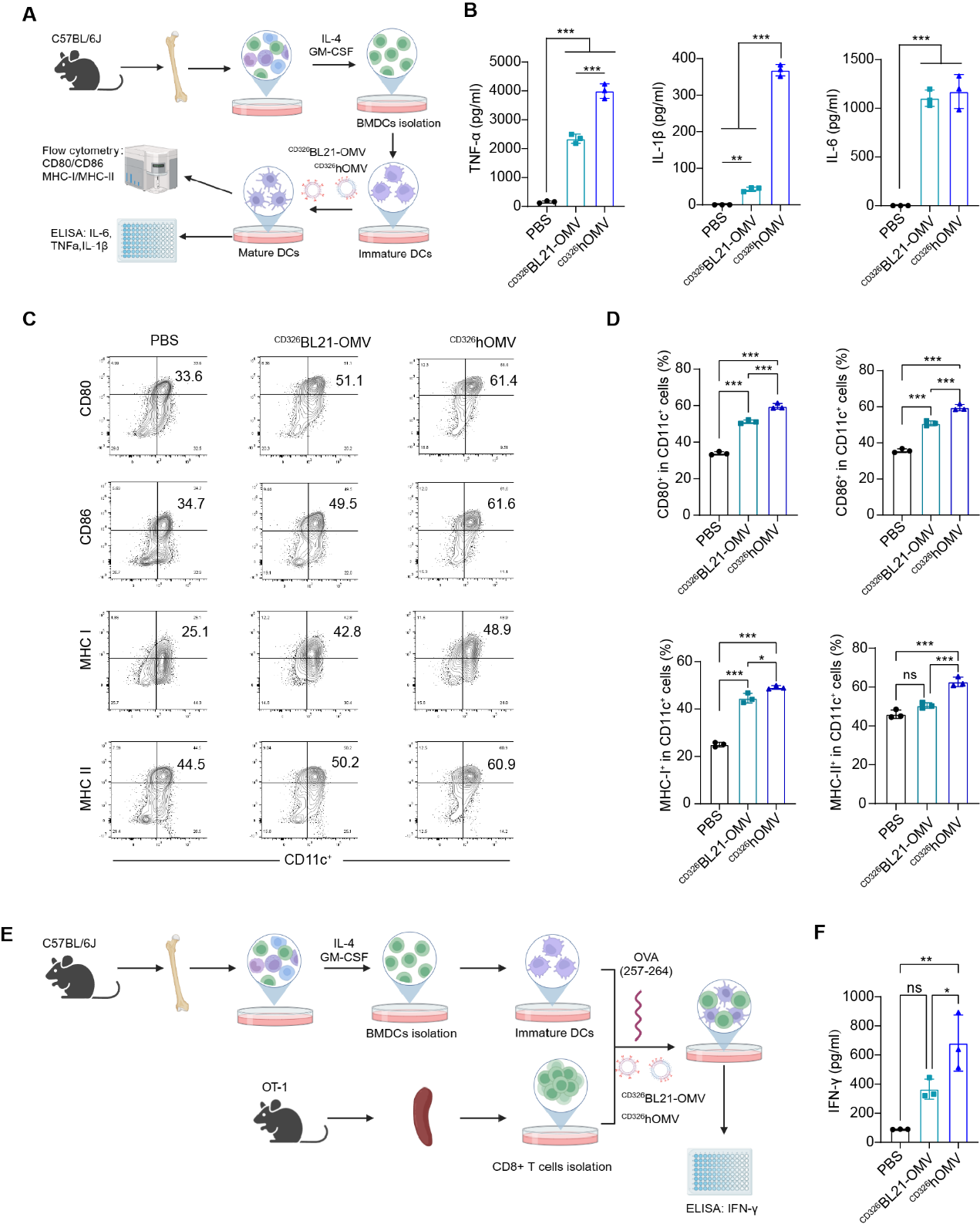


**Figure S5.** Comparison of ^CD326^hOMV and ^CD326^BL21-OMV in activating BMDCs and CD8^+^ T cells. A) Schematic workflow for isolating BMDCs from C57BL/6J mice, followed by analysis of maturation markers and cytokine productions after ^CD326^hOMV or ^CD326^BL21-OMV treatment. B) Pro-inflammatory cytokines (TNF-α, IL-1β, and IL-6) in culture supernatants of BMDCs treated with PBS, ^CD326^BL21-OMV, or ^CD326^hOMV for 12 h, measured by ELISA. C) Representative flow cytometry plots depicting surface expression of maturation markers (CD80, CD86) and antigen-presenting molecules (MHC-I, MHC-II) on CD11c^+^ BMDCs following 24-hour treatment with PBS, ^CD326^BL21-OMV, or ^CD326^hOMV. D) Quantification of CD80^+^, CD86^+^, MHC-I^+^, and MHC-II^+^ populations in CD11c^+^ BMDCs. E) Experimental design for OVA^257-264^-specific CD8+ T cell activation: BMDCs isolated from C57BL/6J mice were treated with OMVs and co-cultured with OVA^257-264^-specific CD8^+^ T cells (isolated from OT-I mice)  in the presence of OVA^257-264^ peptide. F)  IFN-γ production in the supernatant of BMDC-CD8^+^ T cell co-cultures after 24 hours, measured by ELISA. Data are represented as the mean ± SD (n = 3). P values were determined by one-way ANOVA with Tukey’s multiple comparisons test in (B, D, F). NS, not significant. *P < 0.05; **P < 0.01; ***P < 0.001.


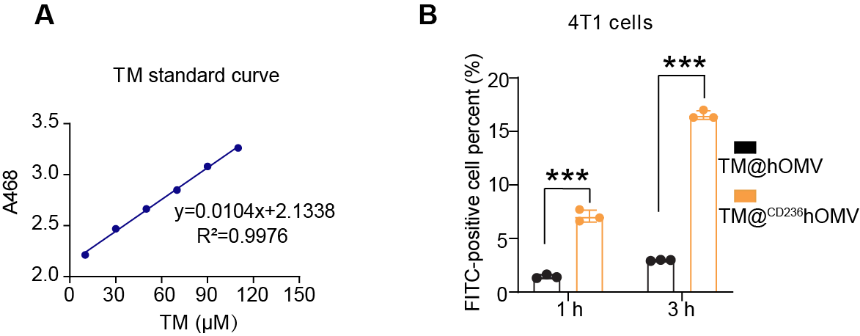


**Figure S6.** Cellular uptake of TM@^CD326^hOMV in 4T1 cells. A) Standard curve of TM. Absorbance values at 468 nm of TM was plotted with the indicated concentration TM. B) Flow cytometry analysis of 4T1 cells incubated with FITC-labeled TM@^CD326^hOMV or TM@hOMV at 37 °C for 1 h or 3 h. Quantitative data are represented as the mean ± SD (n = 3). P values were determined by one-way ANOVA with Tukey’s multiple comparisons test. ***P < 0.001.


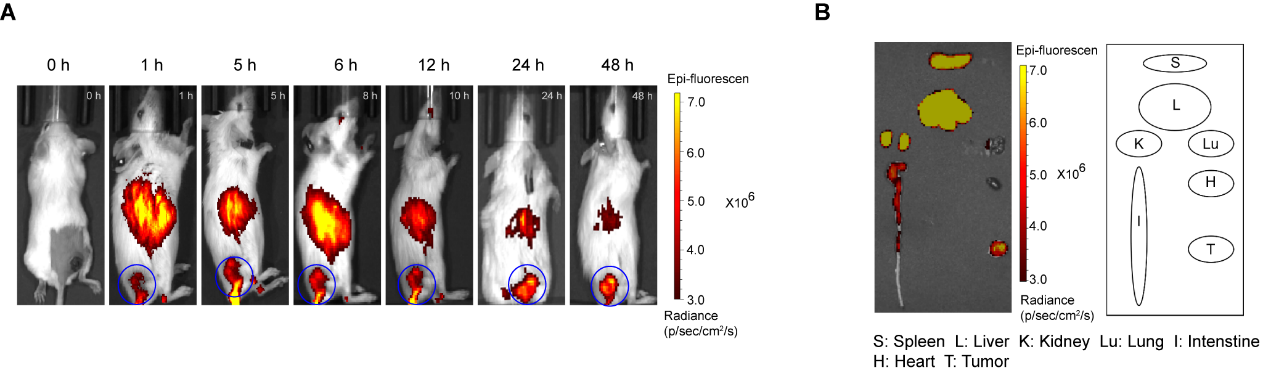


**Figure S7.** *In vivo* biodistribution of TM@^CD326^hOMV. A) *In vivo* fluorescent images of 4T1 tumor-bearing mice at 0, 1, 5, 6, 12, 24 and 48 h post *i.v.* injection of Cy5.5-labled TM@^CD326^hOMV. B) Biodistribution of Cy5.5-labled TM@^CD326^hOMV in spleen, liver, Kindy, lung, heart, intestine, and tumor at 24 h post *i.v.* injection. Representative image is shown from 5 biologically independent mice.


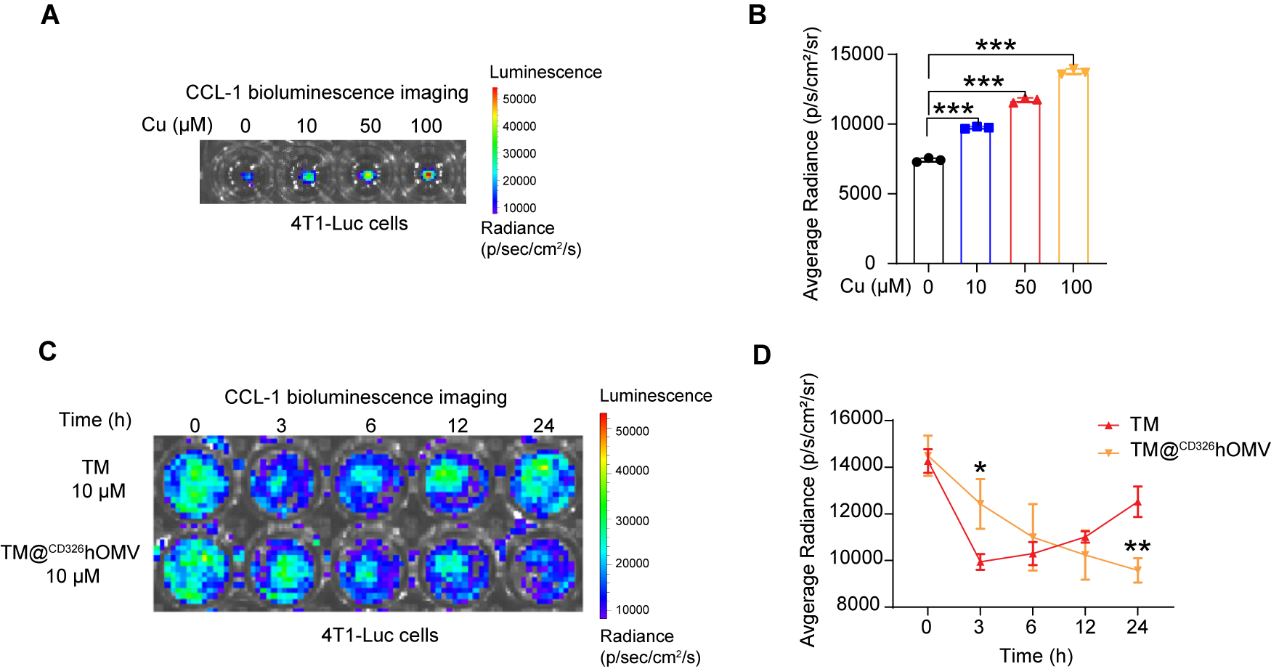


**Figure S8.** *In vitro* copper depletion efficacy of TM@^CD326^hOMV. A,B) CCL-1 measurement of the dynamic changes in Cu^+^ levels in living cells. 4T1-Luc cells treated with the indicated concentration of CuCl_2_ for 12 h and the bioluminescent signals were imaged after the addition of CCL-1 (50 μM) (A). Quantitative analysis of the bioluminescent intensity was shown in (B). C,D) Bioluminescence images of 4T1-Luc cells (C) treated with TM or TM@^CD326^hOMV for indicated time. Quantitative analysis of the bioluminescent intensity was shown (D). P values were determined by one-way ANOVA with Tukey’s multiple comparisons test (B and D). *P < 0.05; **P < 0.01; ***P < 0.001.


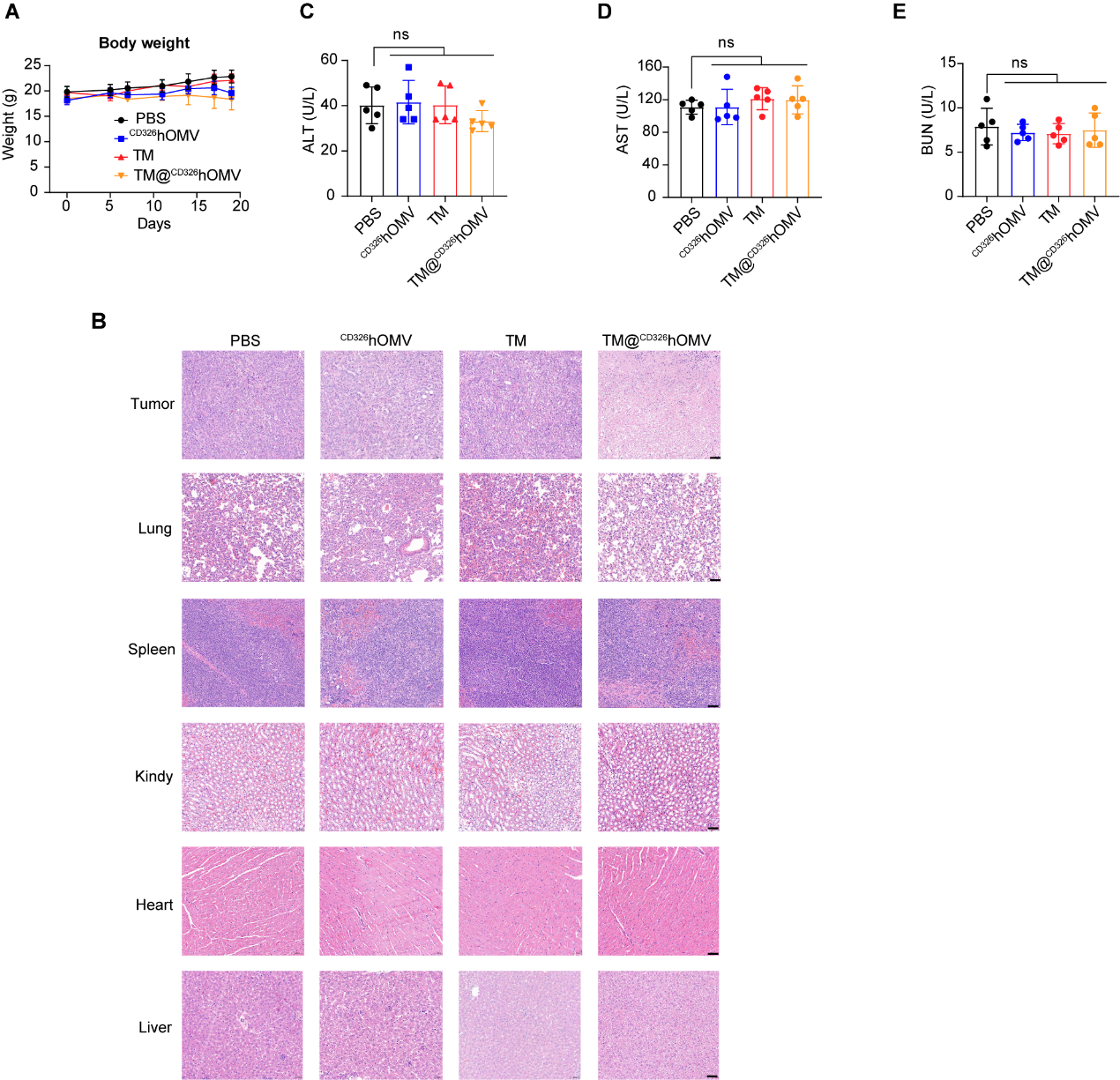


**Figure S9.** Safety assessment of TM@^CD326^hOMV in 4T1 tumor-bearing mice. A) Average body weight of subcutaneously implanted 4T1 tumors treated with PBS, ^CD326^hOMV, TM and TM@^CD326^hOMV. Data are represented as the mean ± SD (n = 5). B) Histological analysis of major organs receiving indicated treatment using H&E staining. Scale bar, 50 μm. C-E) Serum levels of alanine transaminase, ALT (C); aspartate transaminase, AST (D) and blood urea nitrogen, BUN (E). Data are represented as the mean ± SD (n = 5). P values were determined by one-way ANOVA with Tukey’s multiple comparisons test (C, D and E). ns, not significant. *P < 0.05; **P < 0.01; ***P < 0.001.


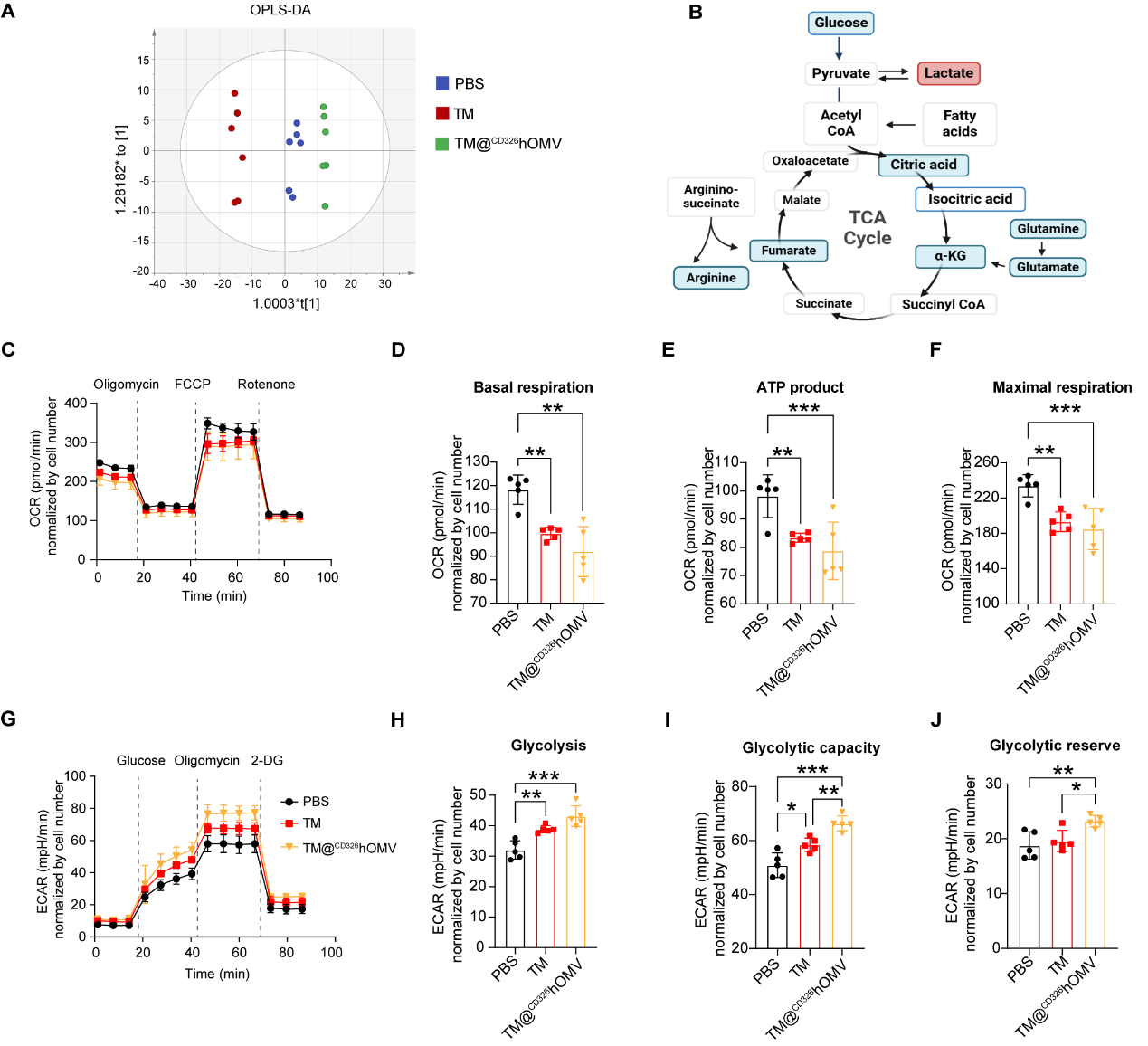


**Figure S10.** TM@^CD326^hOMV induces a metabolic shift from OXPHOS to glycolysis. A) OPLS-DA scores plot of metabolites in the indicated group. B) Schematic representation of several significantly altered metabolites involved in TCA cycle in tumor tissue after TM@^CD326^hOMV treatment. Red box represents the upregulated metabolites comparing TM@^CD326^hOMV group to PBS group, blue box represents the downregulated metabolites, and white box represents metabolite without significant changes. C-F) Seahorse measurement depicting changes in oxygen consumption rate (OCR) (C), basal respiration (D), ATP production (E) and maximal respiration (F) in 4T1 cells treated with 5 μg/ml TM, ^CD326^hOMV and TM@^CD326^hOMV for 12 h, as determined by OCR during a mitochondrial stress test. G-J) Changes in extracellular acidification rate (ECAR) (G), glycolysis (H), glycolytic capacity (I) and glycolytic reserve (J) in ECAR during the glycolysis stress test. Data are represented as the mean ± SD (n = 5). P values were determined by one-way ANOVA with Tukey’s multiple comparisons test in (D-J). *P < 0.05; **P < 0.01; ***P < 0.001.


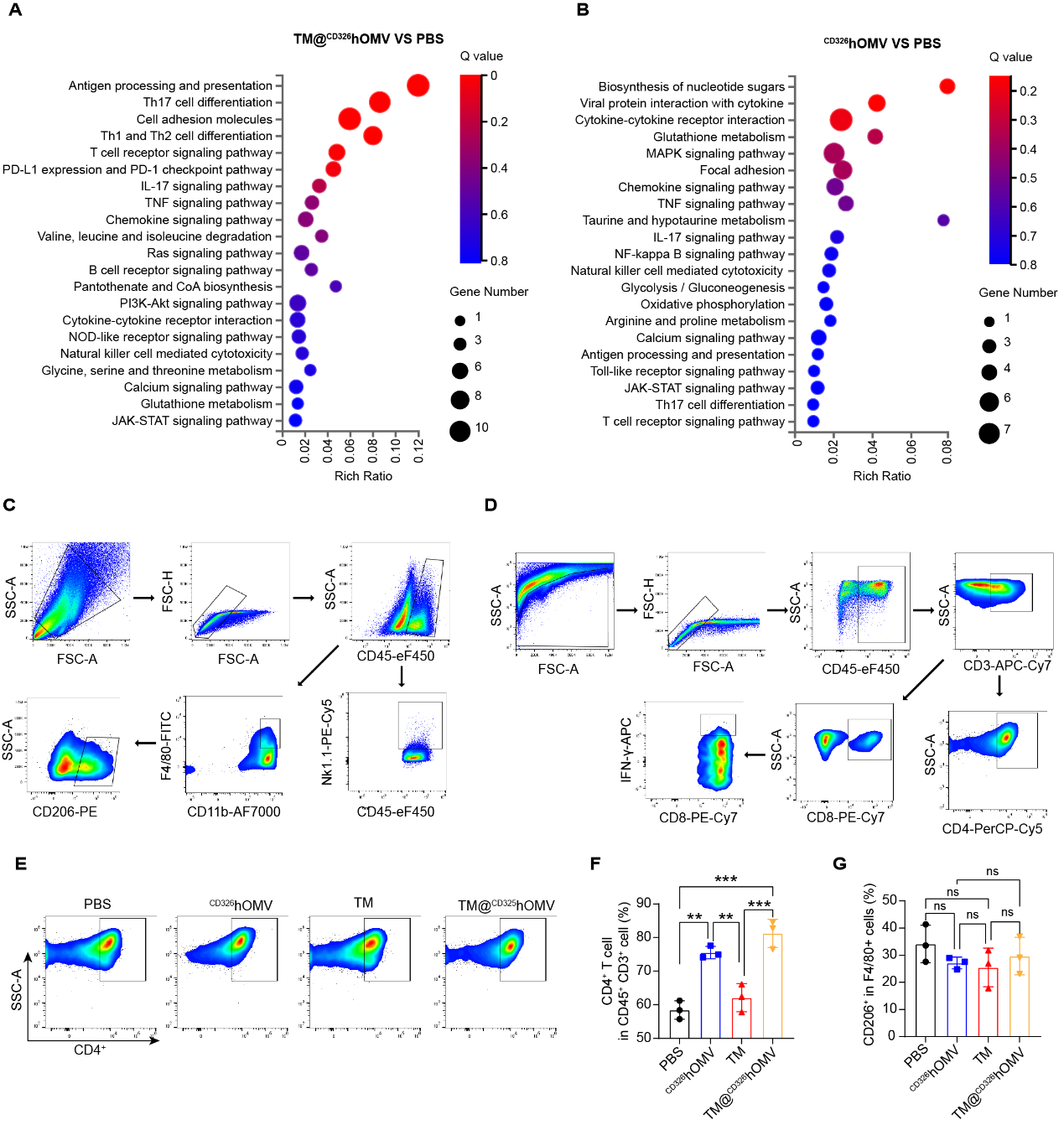


**Figure S11.** TM@^CD326^hOMV reprograms the immune microenvironment in breast tumors. A**,**B) KEGG enrichment of the changed pathways after the treatment of TM@^CD326^hOMV (A) or ^CD326^hOMV (B) compared with PBS. C-D) Gating strategy for flow cytometric analysis of NK cells, CD206^+^ macrophage, CD8^+^ T cells, CD4^+^ T cells and IFN-γ positive CD8^+^ T cells populations in the tumor microenvironment. E-F) Representative flow cytometry dot plots and quantification of CD4^+^ T cells ratio (gated on CD45^+^CD3^+^ cells) in tumor tissues. G) Flow cytometry analysis of CD206^+^ cells within the F4/80^+^ macrophage population across the different treatment groups. Data represent the means ± SD (n = 3) from three independent experiments. P values were determined by one-way ANOVA with Tukey’s multiple comparisons test in F and G. NS, not significant, *P < 0.05; **P < 0.01; ***P < 0.001.


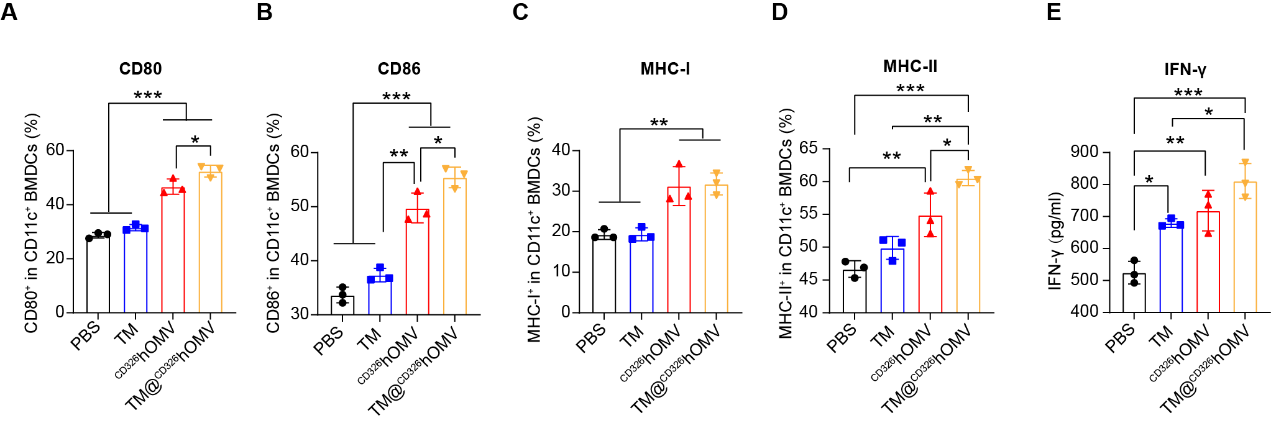


**Figure S12.** TM@^CD326^hOMV promotes dendritic cell maturation and enhances IFN-γ production. A-D) Flow cytometry analysis of dendritic cell (DC) maturation markers, including CD80 (A), CD86 (B), MHC-I (C), and MHC-II (D), in CD11c⁺ bone marrow-derived dendritic cells (BMDCs) after treatment with PBS, TM, ^CD326^hOMV, or TM@^CD326^hOMV. Data are represented as the mean ± SD (n = 3). P values were determined by one-way ANOVA with Tukey’s multiple comparisons test. *P < 0.05; **P < 0.01; ***P < 0.001.


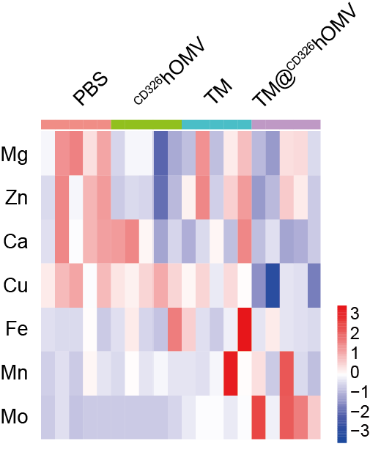


**Figure S13.** ICP-MS analysis of metal levels in the serum of mice treated with PBS, ^CD326^hOMV, TM or TM@ ^CD326^hOMV.
